# Heavy Metal–Pesticide Co-Contamination Under Climate Change: A Systematic Review of Crop Uptake, Food-Chain Transfer, and Sustainable Mitigation

**DOI:** 10.3390/foods15214012

**Published:** 2026-11-09

**Authors:** Mariana Rusu, Irina Gabriela Cara, Emanuel Anti, Ana Ursu, Denis-Constantin Țopa, Gerard Jităreanu

**Affiliations:** 1 Department of Pedotechnics, Faculty of Agriculture, “Ion Ionescu de la Brad” Iasi University of Life Sciences, 3, Mihail Sadoveanu Alley, 700490 Iasi, Romania; mariana.rusu@iuls.ro (M.R.); denis.topa@iuls.ro (D.-C.Ț.); gerard.jitareanu@iuls.ro (G.J.); 2 Research Institute for Agriculture and Environment, “Ion Ionescu de la Brad” Iasi University of Life Sciences, 9, Mihail Sadoveanu Alley, 700490 Iasi, Romania; 3 Department of Oral and Maxillofacial Surgery, EAN Clinic, Private Healthcare Network, 700397 Iasi, Romania

**Keywords:** mixture toxicity, contaminant bioavailability, dietary exposure, edible crops, soil–plant interactions, microbial responses, One Health, sustainable remediation, agroecosystem resilience, contaminant mobility

## Abstract

Heavy metal–pesticide co-contamination is increasingly relevant to food safety because interactions between contaminants can modify their mobility, persistence, bioavailability, and transfer from agricultural soils to edible crops. This systematic review synthesized evidence on the occurrence, environmental behavior, mechanistic interactions, crop uptake, food-chain transfer, and mitigation of metal–pesticide mixtures in agroecosystems under climate-related stress. The evidence synthesized in this review indicates that metal–pesticide co-contamination arises from overlapping agricultural, industrial, and atmospheric inputs. Across the included studies, metal–pesticide co-exposure was associated with synergistic, additive, or antagonistic responses, depending on contaminant identity, concentration, soil properties, and exposure conditions. Co-exposure was associated with altered contaminant mobility, uptake, and accumulation in edible tissues, with implications for dietary exposure and food-chain transfer. The reviewed evidence further indicates that variations in temperature, hydrological conditions, and soil redox dynamics influence contaminant persistence, mobility, transformation, and bioavailability, although these effects remain strongly context-dependent. Biochar, phytoremediation, plant–microbe consortia, and nano-enabled materials show potential to reduce contaminant mobility and crop uptake. Integrated, climate-adaptive monitoring and remediation strategies are needed to better protect soil health, food safety, and the human food chain, while accounting for substantial methodological heterogeneity across available studies.

## 1. Introduction

**Figure 1:**
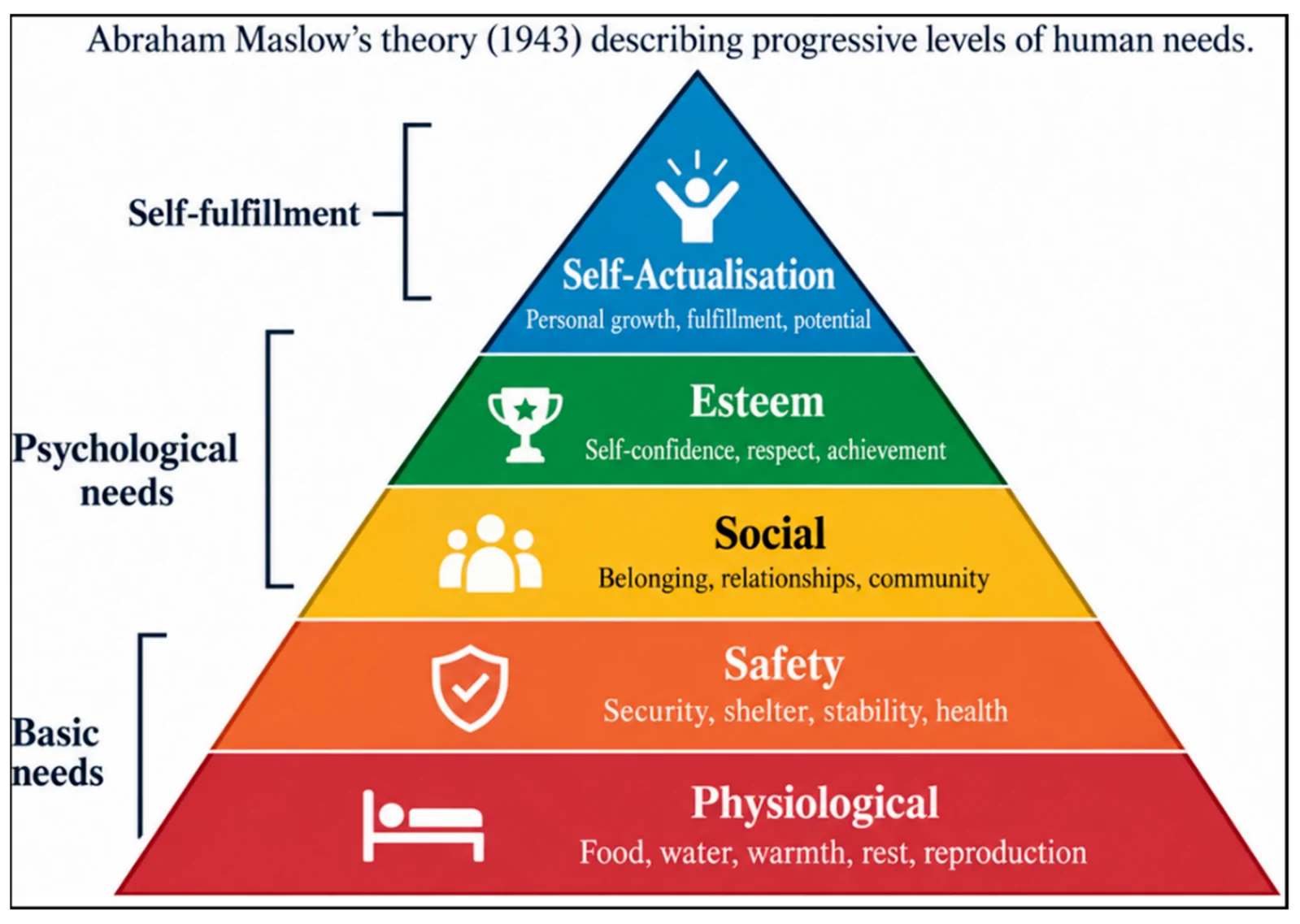
Hierarchical structure of human needs in Maslow’s theory [2].

Food represents one of the most fundamental requirements for human survival and well-being [1]. In Maslow’s hierarchy of needs, food sits at the base of the pyramid as a physiological necessity for sustaining life, along with water, rest, and other essential biological needs (Figure 1) [2]. This conceptual framework emphasizes that access to sufficient food alone is not adequate; food must also be safe, nutritious, and produced within environmental systems capable of sustaining human health [3]. Because agricultural production depends directly on soil quality, the chemical and biological integrity of soils become a critical determinant of food safety. Contamination of agricultural soils with potentially toxic metals and pesticide residues can alter soil physicochemical properties, disrupt microbial and enzymatic processes, impair plant development, and facilitate contaminant transfer into edible plant tissues [4]. Consequently, soil contamination directly links environmental degradation to the integrity of the human food supply, reinforcing the importance of assessing metal–pesticide co-contamination in the broader context of food security, environmental health, and One Health [5].

Human diets draw on both plant and animal food sources, creating multiple pathways through which contaminants in agricultural environments may enter the food chain. Plant roots represent a vital interface between contaminated soils and edible biomass, as they can absorb bioavailable metals and, depending on their physicochemical properties, certain pesticide residues or transformation products, along with water and essential nutrients [6].

After root uptake, contaminants may translocate to shoots, leaves, grains, fruits, and other edible tissues, where they can accumulate to levels relevant to food safety. Contaminated crops and forage can subsequently transfer these substances to livestock, facilitating accumulation within animal tissues and potentially in products such as milk, meat, and eggs. Consequently, humans may be exposed to contaminants from various environmental and trophic sources [7].

**Figure 2:**
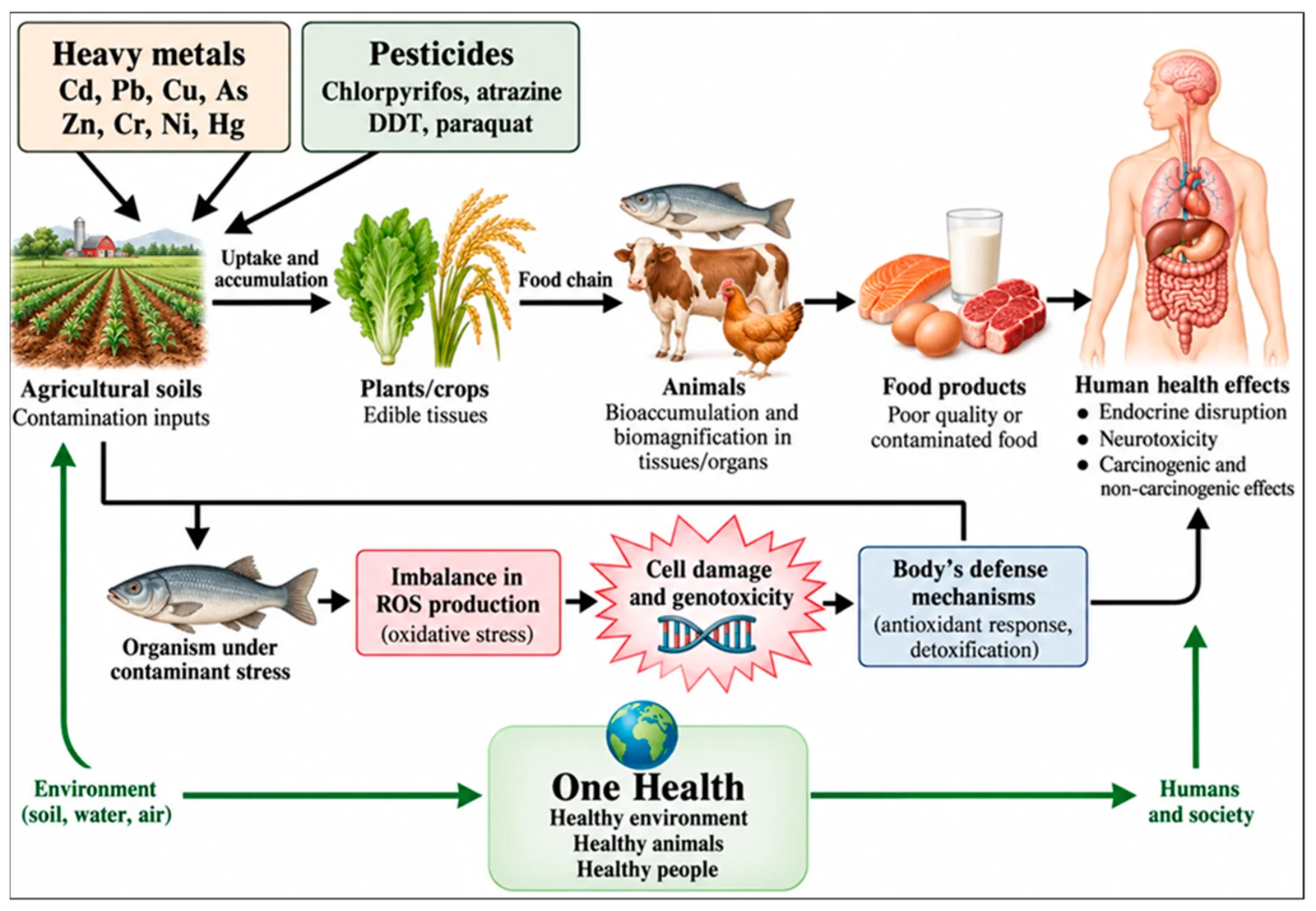
Bioaccumulation and trophic transfer of metal–pesticide co-contaminants.

For persistent, bioaccumulative compounds, these mechanisms can contribute to bioaccumulation within organisms and, under specific trophic conditions, biomagnification throughout food webs. This interconnected soil-plant-animal-human continuum highlights the necessity of an integrated understanding of the ecotoxicological implications of metal-pesticide co-contamination [8]. Understanding the mechanisms underlying contaminant uptake, transformation, accumulation, trophic transfer, and potential adverse effects on food safety and human health is essential (Figure 2).

The widespread co-contamination of agricultural soils with heavy metals and pesticides is a critical environmental challenge of the Anthropocene, affecting ecosystems, food security, and human health globally [9,10]. Intensive agricultural expansion, industrial effluent discharge, and excessive agrochemical use have led to the simultaneous accumulation of inorganic pollutants such as cadmium (Cd), lead (Pb), copper (Cu), and arsenic (As) together with organic pollutants including chlorpyrifos, atrazine, dichloro-diphenyl-trichloroethane (DDT), and paraquat in soils worldwide, with concentrations exceeding regulatory or guideline values for specific contaminants and environmental or food matrices in some regions [11,12]. Studies from selected contaminated agricultural sites in South Asia, sub-Saharan Africa, and parts of Europe have reported Cd concentrations within the mg kg^−1^ (parts per million—ppm) range and, in some cases, Pb concentrations exceeding 100 mg kg^−1^, together with detectable pesticide residues [9,13]. These combined contaminant loads create synergistic stress that profoundly disrupts soil biogeochemistry, crop productivity, food systems, and ecosystem stability.

From a One Health perspective, which recognizes the interconnected health of soil, plants, animals, humans, and the environment, the impacts of heavy metal and pesticide co-contamination extend far beyond the farm boundary. In soils, metals interfere with microbial enzymatic systems, inhibit nutrient cycling, and alter redox dynamics, leading to long-term fertility loss [14]. Plants exposed to such mixtures experience oxidative and hormonal stress, shown by increased reactive oxygen species (ROS) generation, membrane lipid peroxidation, and inhibited photosynthesis [15,16]. These physiological disruptions reduce yields and increase toxic residue accumulation in edible tissues [7]. Through trophic transfer, these contaminants enter livestock and human food chains and bioaccumulate in muscle, liver, and other organs. Chronic exposure has been associated with oxidative DNA damage, endocrine disruption, neurotoxicity, and carcinogenesis [17]. Such interlinked risks exemplify the One Health paradigm: soil degradation compromises plant nutrition, which affects animal and human health, creating a feedback loop that threatens ecosystem resilience and food security [5]. Figure 2 schematically illustrates the transfer of heavy metals and pesticides from contaminated agricultural soils to crops, livestock, food products, and humans, together with key processes of bioaccumulation, biomagnification, oxidative stress, cellular damage, and potential health effects within a One Health framework.

At the environmental level, the persistence of metal–pesticide interactions and associations, stabilized through sorption, chelation, or redox reactions, further complicates remediation [18]. Conventional physicochemical methods, including soil washing and chemical immobilization, can remove pollutants but often exacerbate soil degradation and are economically unviable for diffuse agricultural contamination [19]. Consequently, research has shifted toward eco-remediation and sustainable agricultural practices that use biological, ecological, and agronomic mechanisms to immobilize, transform, or detoxify co-contaminants while maintaining soil health [20].

Although numerous studies have investigated heavy metals or pesticides individually, as well as their effects on soils, plants, microbial communities, and human health, evidence that addressed their combined occurrence and interaction remains heterogeneous. Alengebawy et al. [7] integrated evidence on contaminant sources, accumulation in soils and plants, bioavailability, human-health implications, and synergistic or antagonistic interactions, whereas Shaikh et al. [21] further connected toxicological mechanisms, soil–plant–food transfer, sustainable remediation, and One Health considerations.

Some researchers have addressed metal–pesticide interactions in microbial systems [22], others have explored additive or synergistic phytotoxic effects [23], while additional evidence exists on trophic transfer mechanisms or specific detoxification pathways [21]. However, these studies typically examine only one interaction type, one contaminant pair, or one level of biological organization.

To the best of our knowledge, no previous review has simultaneously integrated the occurrence and co-distribution of heavy metals and pesticides, their chemical and biological interactions, mechanistic effects across soil–plant–microbe–food systems, food-safety implications, and sustainable mitigation strategies within One Health and Planetary Health frameworks [7]. Existing reviews tend to be thematic, organism-specific, contaminant-specific, or geographically limited, leaving major gaps in understanding how multi-contaminant mixtures interact, change across environmental compartments, and potentially enhance toxicity within agroecosystems and food systems, especially under climate-related stressors. This review therefore brings together several dimensions that are often discussed separately. These include the global occurrence and co-distribution of heavy metals and pesticides, the chemical and biological processes that influence their behavior in soils and living organisms, the transfer of contaminants across trophic levels, and the consequences for food safety and human health. It also focuses on sustainable, nature-based remediation approaches from a One Health perspective [24].

Integrating these dimensions is important because metal–pesticide co-contamination can simultaneously affect soil quality, crop production, food safety, ecosystem functioning, and environmental health [25]. A better understanding of these interactions may support earlier identification of soil degradation, improved crop-management strategies under multiple-contaminant stress, a more realistic assessment of mixture effects, and selection of remediation approaches suited to the combined pressures of pollution, climate change, biodiversity loss, and soil degradation [26]. The scope, evidence coverage, methodological framework, and integrated contribution of the present review are summarized in Table 1.

## 2. Materials and Methods

The review used a PRISMA 2020-informed framework [27] for literature identification, screening, and reporting, complemented by a semi-automated computational workflow for thematic classification, eligibility assessment, and evidence prioritization. 

Bibliographic processing and rule-based text analysis were performed in Python 3.13.18 within Google Colab, using Pandas 2.2.3, NumPy 2.1.3, and regular-expression matching. The workflow was designed to distinguish three conceptually separate stages: preliminary screening, empirical eligibility assessment and thematic evidence prioritization. A separate bibliometric dataset was used exclusively for publication-pattern analysis and was not involved in study eligibility or thematic ranking [28].

### 2.1. Eligibility Criteria

The review targeted English-language publications published between 2020 and 2026 addressing metals or metalloids and pesticides in agricultural or food-related systems.

Eligible studies were required to provide evidence to contaminant classes within agricultural or food context and to report at least one outcome that addressed contaminant occurrence, mobility or transformation, physicochemical interactions, soil or microbial responses, plant uptake or toxicity, food-chain transfer, mitigation performance, or environmental or human-health risk.

The eligibility distinguished simple co-detection of metals and pesticides, combined exposure, and experimentally demonstrated mixture interactions. Publications addressing only one contaminant class, reviews, and studies unrelated to agricultural or food-chain systems were excluded from the empirical synthesis.

### 2.2. Information Sources and Search Strategy

The bibliographic search was conducted through the Web of Science platform, using the Web of Science Core Collection and MEDLINE as indexed sources. The search strategy combined four concept groups describing: (i) metals and metalloids; (ii) pesticides; (iii) agricultural and food-chain environments; and (iv) climate-related drivers potentially influencing contaminant behavior. The complete search configuration, search date, time span, database/index selection, and applied filters are provided in Table S1.

Metal-related terms included heavy metals, toxic metals, potentially toxic elements, cadmium, lead, arsenic, copper, zinc, chromium, nickel, and mercury. Pesticide terminology included pesticides, herbicides, insecticides, fungicides, organophosphates, organochlorines, and active substances, including glyphosate, chlorpyrifos, atrazine, and paraquat. Agricultural and food terms included agricultural soil, agroecosystem, cropland, rhizosphere, crop, plant uptake, bioaccumulation, edible tissue, food contamination, food chain, and dietary exposure. Climate descriptors included climate change, global warming, drought, flooding, rainfall, soil moisture, and redox conditions when associated with agricultural, soil, plant, or food-related contexts.

Research restrictions included Food Science and Technology, Chemistry, Agriculture, Biochemistry and Molecular Biology, Plant Sciences, and Environmental Sciences and Ecology. The records retrieved through the Web of Science platform were exported as a consolidated bibliographic dataset. Duplicate checking was performed using Web of Science accession numbers and normalized DOI identifiers, and no duplicate records were detected in the imported dataset.

### 2.3. Screening and Selection Process

Titles, abstracts, author keywords, and Keywords Plus were combined into a searchable bibliographic set. Preliminary screening used dictionaries for metal-related, pesticide, agricultural or food, and outcome-related concepts. Records were assigned to one of three algorithmic decision classes: Include, Review or Exclude.

Evidence of metal–pesticide co-contamination was identified through explicit terminology describing co-contamination, co-exposure, combined pollution, mixtures or joint effects, or through proximity analysis identifying metal- and pesticide-related terms within the same or adjacent sentences.

### 2.4. Data Collection and Extraction

For each study, investigated metals and metalloids and pesticide classes or individual active substances were recorded together with their reported concentrations in soils, plants, water, or food-related matrices. Analytical procedures, including ICP-MS, AAS, GC-MS, LC-MS/MS and HPLC, were documented where available.

Extracted outcomes included contaminant mobility and transformation, physicochemical interactions, soil and microbial responses, plant uptake and physiological effects, accumulation in edible tissues, food-chain transfer, dietary exposure and environmental or human-health risk indicators.

### 2.5. Thematic Relevance and Quality Control

Thematic relevance was assessed using weighted keyword dictionaries specifically developed for the eight evidence domains. Keyword weights ranged from 2 to 7 according to thematic specificity. Highly specific descriptors directly related to contaminant transfer, exposure, or mitigation received the highest weights, whereas broader agricultural or environmental terms received lower weights.

For each publication, titles, abstracts, author keywords, and Keywords Plus were combined into a searchable text corpus. A weighted relevance score was calculated independently for each thematic category:

(1) $$S_{ij}=\sum_{k=1}^{m_{j}}w_{jk}I_{ijk}$$

where: $S_{ij}$ represents the relevance score of publication *i* in thematic category *j*, $w_{jk}$ is the predefined weight of keyword *k*, and $I_{ijk}$ equals 1 when the keyword is identified and 0 otherwise. Each keyword contributed only once per publication, regardless of its frequency.

The complete category-specific keyword dictionaries and numerical weights are provided in Table S2. The weighted scores quantify thematic relevance and were not interpreted as methodological-quality or risk-of-bias scores.

### 2.6. Screening Bias and Selection Error Assessment

Potential screening errors were considered throughout the computational workflow. Keyword-based classification can be affected by terminology variation, ambiguous word matches, incomplete abstracts, and differences in indexing between publications. The Review category was therefore retained during preliminary screening to minimize exclusion of potentially relevant but incompletely described studies.

The extended eligibility audit further distinguished explicit co-contamination terminology from contaminant proximity within the text. Metal- and pesticide-related terms occurring within the same or adjacent sentences were considered evidence warranting inclusion at the abstract-screening stage when the agricultural or food-related context and empirical outcome criteria were also satisfied.

The initial Top-200 procedure was used as a broad thematic retrieval step rather than as a final eligibility criterion. Because thematic scoring may still underrepresent studies using uncommon terminology, the resulting prioritization should be interpreted as a structured evidence-selection procedure rather than as a quantitative estimate of screening sensitivity or specificity.

No formal estimate of false-negative screening probability was calculated.

### 2.7. Thematic Ranking and Evidence Prioritization

Following eligibility assessment, the 534 Eligible or full-text-checked publications were ranked independently within each thematic category. A composite category-specific relevance score was calculated by combining the weighted thematic score with predefined indicators of stronger preliminary relevance, evidence of metal–pesticide co-occurrence and multidisciplinary thematic coverage:

(2) $$R_{ij}=S_{ij}+3I_{strong}+2I_{CoContam}+I_{multi}$$

where: $R_{ij}$ represents the composite ranking score of publication (i) in thematic category (j), and $\left(S_{\left\{ij\right\}}\right)$ is the weighted thematic relevance score.

The binary indicator $I_{strong}$ equals 1 when a publication was classified as a Strong candidate during the extended screening stage and 0 otherwise.

The binary indicator $I_{CoContam}$ equals 1 when explicit or proximity-based evidence of metal–pesticide co-contamination was identified and 0 otherwise.

The binary indicator $I_{multi}$ equals 1 when a publication was associated with more than one thematic category and 0 otherwise.

Category-specific thresholds were instead defined from the empirical score distributions. Publications at or above the 85th percentile (Q85) were retained, and all records tied at the threshold were preserved.

### 2.8. Sensitivity Analysis of Evidence Prioritization

To evaluate the robustness of the percentile-based evidence prioritization, a sensitivity analysis was performed using alternative category-specific thresholds at the 80th, 85th, and 90th percentiles (Q80, Q85, and Q90). The Q85 configuration was treated as the reference. Stability was assessed using the number of thematic assignments and unique publications, the proportion of Q85 publications retained under alternative thresholds, Jaccard similarity between unique-publication sets, and domain-specific retention. All records tied at the respective percentile thresholds were retained.

### 2.9. Evidence Synthesis

Evidence was synthesized narratively within the eight thematic domains. Quantitative outcomes were retained as reported in the original studies, including contaminant concentrations, uptake and translocation indices, degradation or removal efficiency, biological-response measures and food-safety or dietary-risk indicators.

Because of substantial heterogeneity in contaminant combinations, matrices, experimental designs, analytical methods, exposure conditions and reported outcomes, no pooled effect size or meta-analysis was applied. Co-occurrence of metals and pesticides was distinguished from experimentally demonstrated interaction effects. Claims of synergistic, antagonistic or additive responses were considered only when supported by appropriate comparison groups or experimental contrasts.

### 2.10. Certainty Assessment

Confidence in the evidence was assessed using a structured descriptive evidence framework rather than a formal GRADE approach, because of the heterogeneity of the included experimental, field, monitoring, mechanistic, modeling, and review studies. Evidence was evaluated according to five dimensions: (i) directness of metal–pesticide co-exposure or interaction; (ii) study design and replication; (iii) analytical reliability and contaminant confirmation; (iv) environmental and food-chain relevance; and (v) consistency and applicability of the reported findings.

Interpretive weight was assigned to studies examining metal–pesticide combinations, using appropriate comparison or control groups, analytically confirmed contaminants, and employing replicated experimental or field designs. Direct metal–pesticide interaction evidence was distinguished from simple co-occurrence, mechanistic supporting evidence, and contextual or indirect evidence.

For food-safety interpretation, direct measurements in food matrices and studies reporting dietary-exposure or risk endpoints were distinguished from evidence from soils, environmental matrices, or non-edible tissues. Evidence was classified as high, moderate, or supporting. High-strength evidence required direct metal–pesticide co-exposure or interaction with a directly relevant food, dietary exposure, or risk endpoint.

Moderate-strength evidence included either direct metal–pesticide studies without a direct food endpoint or analytically confirmed co-occurrence in food-related matrices accompanied by exposure or risk-relevant outcomes.

Records with insufficient information for unambiguous classification were subjected to manual verification, whereas retracted publications were excluded from evidence interpretation. No formal GRADE assessment, pooled certainty score, or combined relevance–quality index was applied.

### 2.11. Bibliometric Mapping and Analysis

Bibliometric mapping was conducted using a separate dataset and was analytically independent of the systematic-review screening and prioritization workflow.

VOSviewer (version 1.6.21) was used to examine publication trends, keyword co-occurrence, citation networks, and thematic clusters. Bibliometric indicators were used only to characterize the development and structure of the research field and did not contribute to eligibility decisions, weighted thematic scores, or Q85 evidence prioritization.

## 3. Research Literature

### 3.1. Study Selection

**Figure 3:**
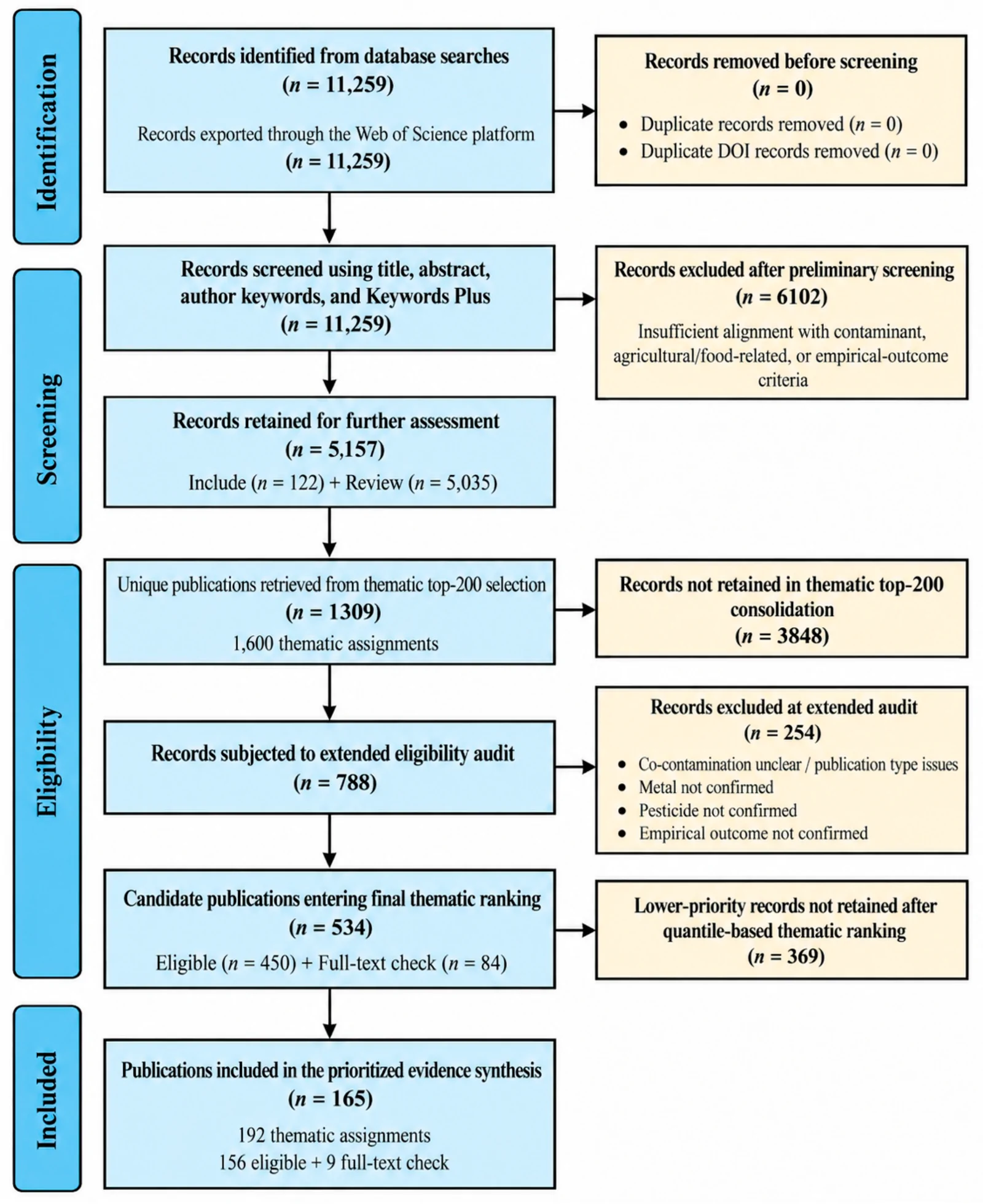
PRISMA 2020 flow diagram showing the identification, screening, eligibility assessment, and inclusion of studies in the systematic review of heavy metal–pesticide co-contamination in agricultural and food-production systems.

The study-selection process followed the PRISMA 2020 framework and was complemented by a structured algorithm-assisted procedure designed for the multidisciplinary evidence linking metal contamination, pesticide occurrence, agricultural systems, crop uptake, and food-chain transfer. The bibliographic dataset used for systematic-review screening was retrieved through the Web of Science platform, using the Web of Science Core Collection and MEDLINE as indexed sources. All records were exported through the Web of Science interface and processed as a consolidated bibliographic dataset. The complete search configuration, search date, time span, database/index selection, and applied filters are provided in Table S1. Compliance with the PRISMA 2020 reporting framework is documented in the PRISMA 2020 Checklist provided as Table S2, while the study identification, screening, eligibility, and evidence-prioritization workflow is summarized in the PRISMA flow diagram (Figure 3).

The search architecture combined four principal groups of terms. The first group described metals and metalloids, including heavy metals, toxic metals, potentially toxic elements, cadmium, lead, arsenic, copper, zinc, chromium, nickel, and mercury. The second group targeted pesticide-related terminology, including pesticides, herbicides, insecticides, fungicides, organophosphates, organochlorines and representative active substances such as glyphosate, chlorpyrifos, atrazine and paraquat. A third group defined the agricultural and food-chain context through terms such as agricultural soil, agroecosystem, cropland, rhizosphere, crop, plant uptake, bioaccumulation, edible tissue, food contamination, food chain and dietary exposure. Climate descriptors, including climate change, global warming, drought, flooding, rainfall, soil moisture and redox conditions, were incorporated where they occurred in conjunction with agricultural, soil, plant or food-related terms. Equivalent Boolean combinations were adapted to the syntax and indexing structure of each database.

The initial search generated 11,259 records. Of these, 11,198 contained abstract information, whereas 61 records lacked an abstract. No duplicate Web of Science accession numbers or normalized DOI records were identified in the consolidated dataset.

Title- and abstract-based screening was subsequently performed using three decision categories—Include, Review and Exclude—defined according to the degree of alignment between contaminant identity, agricultural or food-chain context and empirical relevance.

Records were classified as Include when the title and abstract showed strong evidence of relevance to metal–pesticide contamination and contained a clear agricultural, crop, soil or food context together with a relevant environmental, biological or food-safety outcome.

Records were assigned to Review when they retained relevance, but the information available in the title and abstract was insufficient to confirm one or more elements, particularly contaminant co-occurrence, agricultural or food-chain applicability, or the presence of an empirical outcome.

Records were classified as Exclude when they lacked sufficient alignment with the predefined search concepts, contained no relevant empirical outcome, or represented publication types outside the predefined eligibility framework.

Application of these rules resulted in 122 Include records, 5035 Review records, and 6102 Exclude records. Because the Review category was designed to preserve relevant described studies, Include and Review records were retained for thematic assessment, which retained a spectrum of 5157 publications.

The retained records were then evaluated against eight predefined thematic categories: Sources, Crop Uptake, Physicochemical Interactions, Plant Responses, Microbial Responses, Food Safety, Climate and Mitigation. Weighted thematic scores were calculated from titles, abstracts, author keywords, and Keywords Plus using category keyword dictionaries. Using unique publication identifiers, this procedure yielded 1309 unique publications, including 1053 associated with a single thematic category and 256 contributing to more than one category (Table 2).

The 1309 publications were subsequently subjected to an eligibility audit. Records were retained for this audit when they fulfilled at least one of three predefined conditions: (i) evidence of metal and pesticide occurrence together with an agricultural or food context and an empirical outcome; (ii) evidence of at least one contaminant class together with an agricultural or food context and a maximum thematic score of at least 8; or (iii) multidisciplinary thematic relevance, defined by assignment to more than one thematic category, together with an agricultural or food context and a maximum thematic score of at least 8. Application of these criteria retained 788 publications for extended eligibility assessment, whereas 521 records did not advance to this stage.

Because individual publications could simultaneously address several components of the contamination pathway, studies were allowed to contribute to more than one thematic category. The thematic classification was therefore treated as non-mutually exclusive during ranking, while unique Web of Science accession identifiers were used to quantify overlap and consolidate the final evidence corpus.

The complete study-selection procedure is summarized in the PRISMA 2020 flow diagram (Figure 3).

### 3.2. General Research Context

#### 3.2.1. Topics and Directions Related to Agroecosystem Co-Contamination

**Figure 4:**
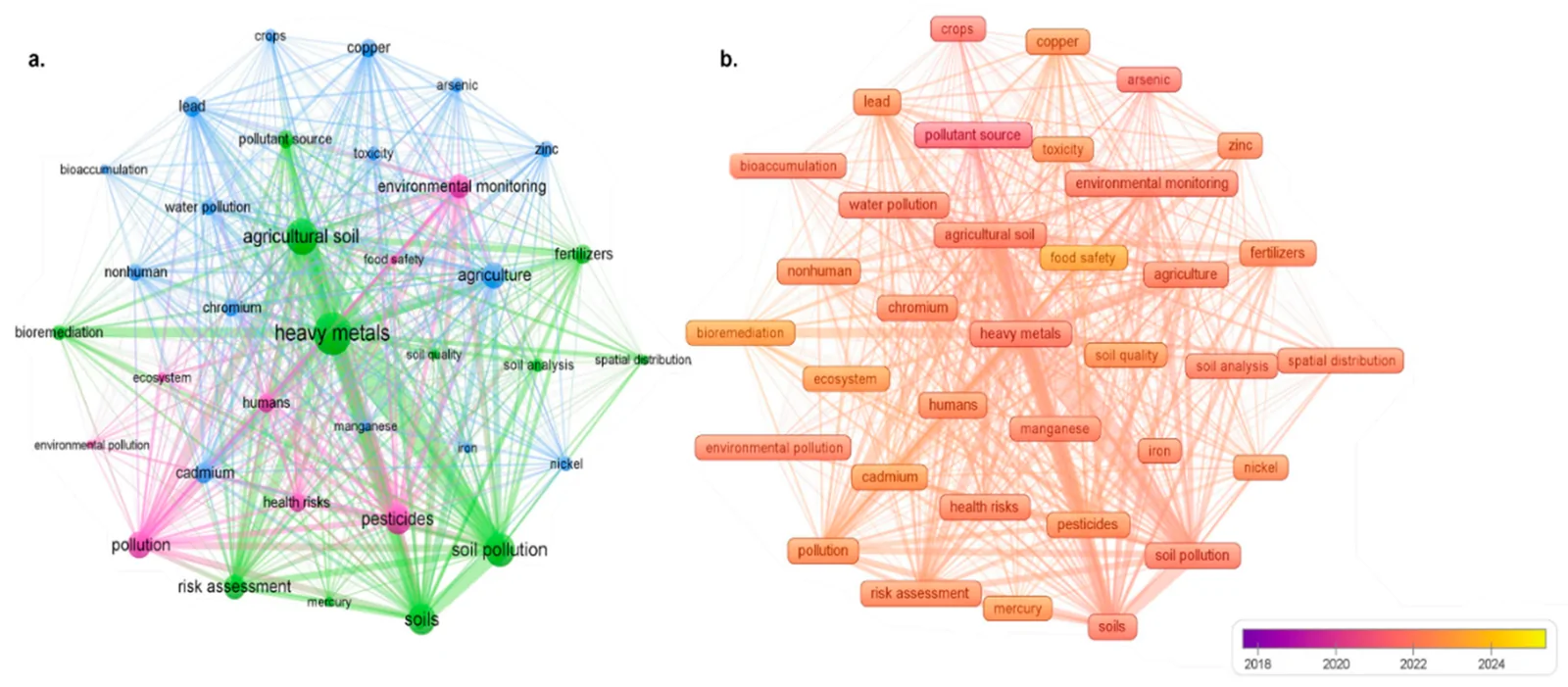
VOSviewer-Based Keyword Co-Occurrence Network (**a**) and Temporal Overlay Visualization (**b**) for Publications on Heavy Metal–Pesticide Co-Contamination in Agroecosystems (2018–2025).

Keyword co-occurrence mapping using VOSviewer enables the visualization of dominant research themes, the semantic proximity between concepts, and the temporal evolution of scientific interest within a field. Larger nodes represent high-frequency terms, while stronger associations between concepts appear as thicker connecting lines [25]. The temporal overlay visualization further highlights how research priorities have shifted over time, with colors indicating the approximate year when keywords gained prominence [26,28] (Figure 4).

Figure 4a illustrates three major thematic clusters emerging from the co-occurrence network:

Cluster 1. Agricultural Soil Pollution and Risk Pathways (Green Cluster)—This central cluster comprises 11 interrelated terms focusing on the contamination of agricultural soils with heavy metals and pesticides, their spatial distribution, and the resulting environmental and food-chain risks. Strong linkages connect heavy metals, agricultural soils, soil pollution, bioaccumulation, bioremediation, toxicity, and risk assessment, indicating that much of the literature centers on quantifying contamination levels and evaluating ecological and human exposure pathways.

Cluster 2. Trace Elements, Toxicity and Bioaccumulation (Orange Cluster)—The most extensive cluster, containing 16 keywords, is dominated by essential and non-essential trace elements, including iron, zinc, cadmium, manganese, nickel, arsenic, chromium, copper, and lead, and their ecological and physiological implications. Terms such as agriculture, crops, water pollution, and soil quality reinforce the multidisciplinary intersections between plant physiology, environmental chemistry, and agricultural management. This cluster reflects research focused on the toxicity, transport, bioaccumulation, and behavior of mixed contaminants in soils and crops.

Cluster 3. Pesticides, Ecosystem Health and Monitoring (Purple/Pink Cluster)—This group integrates keywords related to pesticide contamination, ecosystem risks, and monitoring practices. Concepts such as ecosystem, humans, environmental monitoring, and health risks highlight the societal relevance of pesticide-related hazards and the need for robust surveillance systems. The presence of pesticide-related terms in a distinct cluster underscores the growing attention given to monitoring and mitigating their impacts within One Health and Planetary Health frameworks.

Figure 4b presents the temporal overlay of keywords, revealing a gradual shift in research priorities over the last decade. Earlier studies (2018–2020), represented by cooler colors (purple/blue), primarily addressed general pollution sources, baseline contamination levels, and heavy metal distribution in soils. Subsequent studies (2021–2022), shown in intermediate colors, increasingly focused on monitoring specific elements, pesticide residues, and ecological risk evaluations.

Across the final set of 165 publications, article prevalence analysis showed that pesticide terminology was the most widespread in the selected 117 studies (70.9%). Toxicity-related concepts were identified in 46 publications (27.9%), followed by heavy metals in 44 (26.7%), risk assessment in 28 (17.0%), and agricultural soils in 25 (15.2%). Food-safety terminology occurred in 22 studies (13.3%), while bioaccumulation was identified in 21 (12.7%).

More specialized research directions were represented by rhizosphere processes (15 studies; 9.1%), bioremediation (14; 8.5%), and climate change (13; 7.9%). Dietary exposure and phytoremediation were each identified in eight publications (4.8%), whereas drought and soil contamination appeared in seven studies each (4.2%). Explicit references to food-chain transfer were less frequent (6 studies; 3.6%), while environmental monitoring and soil pollution each occurred in four publications (2.4%).

#### 3.2.2. Geographical Distribution and International Collaboration Patterns

**Figure 5:**
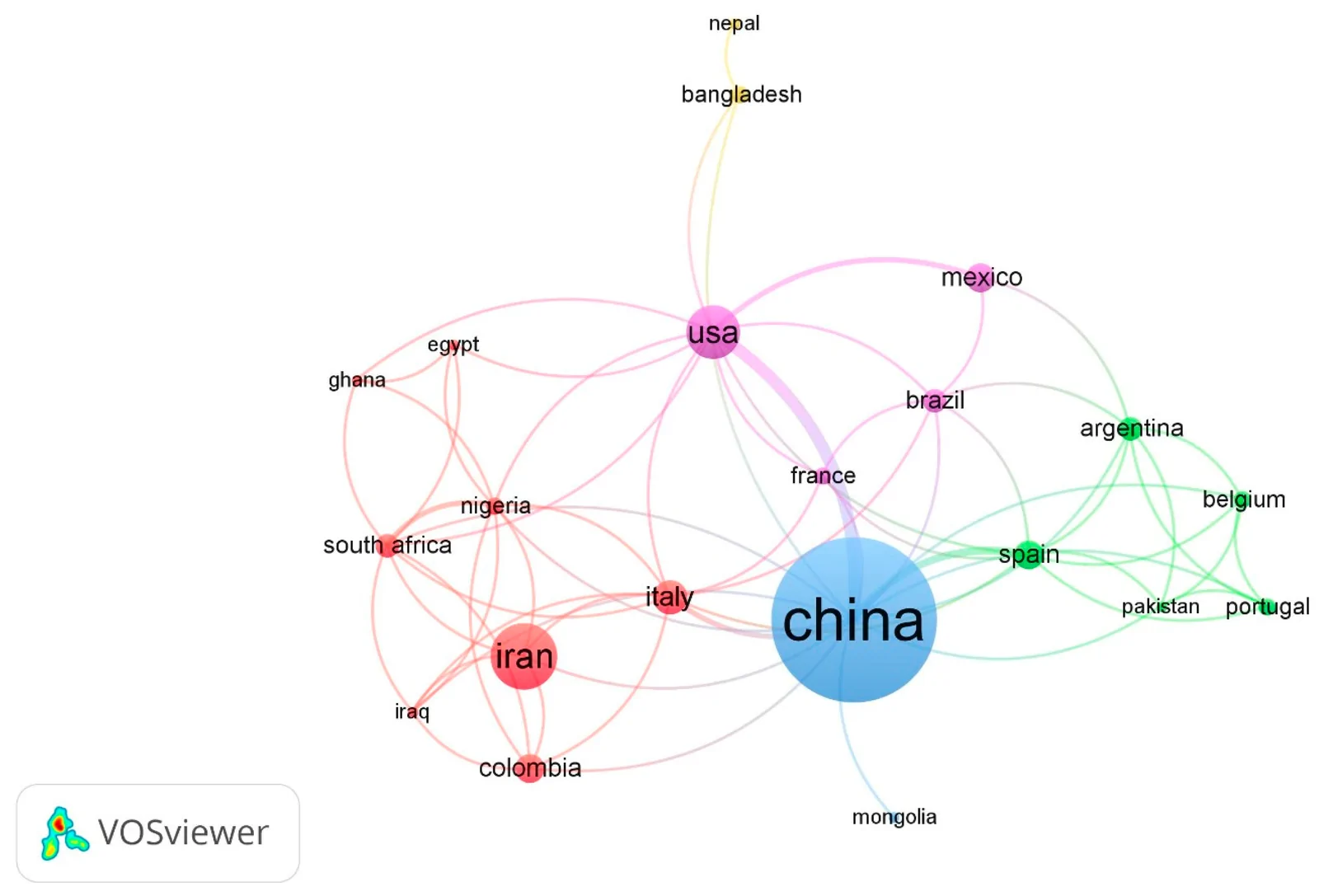
Geographical Distribution and International Collaboration Patterns.

The co-authorship network generated through VOSviewer highlights the geographical distribution of scientific contributions and the extent of international collaborations in research on heavy metal-pesticide co-contamination in agroecosystems. As illustrated in Figure 5, China occupies a central and highly influential position within the global research network, ranking first in terms of publication output and exhibiting 40 co-occurrences. China also demonstrates a high level of international integration, with 16 collaborative links, the strongest being with the United States and Spain, reflecting well-established transcontinental partnerships in environmental science and agricultural pollution research.

Following China, other major contributors include Iran, the United States, Italy, South Africa, and Spain, each forming significant regional hubs. These countries display dense intra- and inter-regional connections, indicating active scientific exchanges and shared research priorities regarding contaminant behavior, ecological impacts, and mitigation strategies.

The network reveals four major regional clusters:

- Asia (blue color), dominated by China and supported by active contributors such as Iran and Pakistan;
- Middle East-Africa (red color), with strong representation from Iran, Egypt, South Africa and Nigeria;
- Europe (green color), including Spain, Belgium, Portugal and Italy, which exhibits high internal connectivity;
- The Americas (purple color), represented by the USA, Brazil, Mexico, Argentina and Colombia.

The varying thickness of the connecting lines reflects the strength of collaborative ties, with transregional partnerships, particularly China-USA and Spain-Brazil, playing a key role in shaping global research directions.

Overall, the collaboration network underscores the highly internationalized nature of research on agroecosystem co-contamination, while also revealing regional asymmetries that shape the production and dissemination of knowledge in this rapidly expanding domain.

### 3.3. Thematic Mapping and Final Evidence Prioritization

#### 3.3.1. Eligibility and Exclusion Criteria

Eligibility assessment was conducted independently of thematic ranking (Table 3). Publications were considered eligible when they provided evidence of metal or metalloid contamination and pesticide occurrence within an agricultural, crop, soil, or food-related context and reported an empirical environmental, biological, or food outcome. Evidence of co-contamination was accepted when the title or abstract contained explicit terminology that described combined contamination, co-exposure, mixtures or joint effects, or when metal- and pesticide-related terms occurred within the same or adjacent sentences.

Records were assigned to Full-text check when the required contaminant, context, and outcome components were present, but the abstract did not provide sufficient information to confirm co-contamination unequivocally. Publications were excluded when they addressed only one contaminant class, lacked an agricultural or food-related context, did not report a relevant empirical outcome, or represented an ineligible publication type such as a review, editorial, meeting abstract, or other non-original research format.

Application of these criteria to the extended audit corpus resulted in 450 Eligible publications, 84 Full-text check records, and 254 Exclude records. This approach retained uncertain but potentially relevant studies for additional assessment rather than excluding them solely on the basis of incomplete abstract information.

#### 3.3.2. Thematic Relevance and Ranking

The 534 retained publications were ranked independently within the eight thematic categories using weighted thematic relevance scores. Composite ranking incorporated the thematic score together with additional indicators of strong preliminary relevance, co-contamination evidence, and multidisciplinary thematic coverage.

Because score distributions differed among thematic categories, category-specific thresholds were derived from the empirical distribution of the composite relevance scores rather than applying a common numerical threshold or a fixed number of publications per category. Publications at or above the 85th percentile (Q85) were retained, while tied scores at the threshold were preserved to avoid arbitrary exclusion of studies with identical relevance scores.

The resulting Q85 thresholds were 12 for Sources, 11 for Crop Uptake, 11 for Physicochemical Interactions, 14 for Plant Responses, 12 for Microbial Responses, 19 for Food Safety, 14 for Climate and 12 for Mitigation.

Application of these thresholds retained 192 thematic assignments: 16 for Sources, 27 for Crop Uptake, 31 for Physicochemical Interactions, 41 for Plant Responses, 17 for Microbial Responses, 26 for Food Safety, 12 for Climate and 22 for Mitigation.

#### 3.3.3. Sensitivity Analysis of Q85 Prioritization

Sensitivity analysis (Table 4) indicated that the Q85 prioritization represented an intermediate and comparatively stable configuration. Lowering the threshold to Q80 increased the evidence corpus from 165 to 193 unique publications while retaining all Q85-selected studies (100% retention; Jaccard similarity = 0.855). Increasing the threshold to Q90 reduced the corpus to 116 unique publications and retained 70.3% of the Q85 set (Jaccard = 0.703).

Domain-specific analyses showed complete stability across the tested thresholds for Crop Uptake and Mitigation. Sources and Climate were also fully retained at Q90, whereas Physicochemical Interactions and Plant Responses were more sensitive to the stricter threshold, retaining 32.3% and 41.5% of their respective Q85 selections. Microbial Responses and Food Safety showed intermediate retention of 58.8% and 69.2%, respectively.

#### 3.3.4. Final Evidence Corpus and Thematic Distribution

After consolidation using unique Web of Science accession identifiers, the 192 thematic assignments corresponded to 165 unique publications. Of these, 138 publications were associated with a single thematic category, whereas 27 publications contributed to more than one category.

Within this Q85-prioritized corpus, 156 publications had already satisfied the eligibility criteria based on title and abstract information, while nine publications required additional full-text verification. Consequently, the definitive evidence corpus will comprise between 156 and 165 publications, depending on the outcome of the final full-text assessment of these nine records (Table 5).

The presence of 27 multi-category publications reflects the interconnected nature of metal–pesticide co-contamination research, particularly where contaminant interactions, crop uptake, plant responses, food-chain transfer, climate-related processes and mitigation strategies are investigated within the same experimental framework.

Records entering category ranking represent Eligible and Full-text check publications with a positive thematic score in the corresponding category. Q85 composite-score cutoff represents the category-specific 85th-percentile threshold. All records with scores equal to or greater than the threshold were retained; therefore, tied scores may result in more than 15% of records being selected. Individual publications could contribute to more than one thematic category.

#### 3.3.5. Evidence-Strength Assessment

The descriptive evidence-strength assessment showed that only a limited subset of the final corpus provided direct evidence of metal–pesticide co-exposure or interaction. Of the 165 unique publications retained, 12 studies were manually confirmed as direct metal–pesticide evidence, whereas 86 documented co-occurrence, 27 provided mechanistic supporting evidence, 29 were contextual or indirect, and 8 were review-based supporting studies. Two records required additional manual verification, while one retracted publication was excluded from evidence interpretation.

Based on the predefined evidence-strength criteria, 2 studies (1.2%) were classified as high-strength evidence, 31 (18.8%) as moderate-strength evidence, and 129 (78.2%) as supporting evidence. Among the 31 moderate-strength studies, 10 provided direct metal–pesticide evidence without a directly relevant food endpoint, whereas 21 documented analytically confirmed co-occurrence in food-related matrices together with exposure- or risk-relevant outcomes.

The distribution of evidence strength varied across thematic domains. High-strength evidence was identified only in the Food Safety and Plant Responses domains. Moderate-strength evidence was most frequent in Food Safety (n = 21) and Physicochemical Interactions (n = 5), with smaller contributions from Crop Uptake (n = 1), Mitigation (n = 1), Plant Responses (n = 1), and Sources (n = 2). The Climate domain consisted entirely of supporting evidence, while most studies in the remaining domains were also classified as supporting. Overall, these findings indicate that the literature addressing metal–pesticide contamination is substantially broader than the subset of studies directly demonstrating combined exposure or interaction effects.

### 3.4. Occurrence and Environmental Behavior of Co-Contaminants

#### 3.4.1. Sources and Pathways of Heavy Metals and Pesticides in Agroecosystems

The co-occurrence of heavy metals and pesticides in agroecosystems reflects the superposition of agricultural inputs, natural geochemical processes, atmospheric deposition, irrigation practices, and industrial activities. Source-apportionment studies consistently indicate that potentially toxic elements in agricultural soils rarely originate from a single pathway, but, rather, from combinations of geogenic and anthropogenic inputs whose relative contribution varies among locations and land-use systems [29,30].

Agricultural inputs represent an important pathway for both contaminant classes. Mineral fertilizers, particularly phosphate-based fertilizers, can contribute Cd and other trace elements, while pesticide application may directly introduce organic contaminants and may also contribute Cu and other metals when metal-containing plant-protection products are repeatedly applied. In greenhouse vegetable soils, source apportionment attributed substantial proportions of heavy-metal contamination to fertilizer input, pesticide spraying, coal combustion, and plastic-film-related agricultural activities [31]. Similarly, studies conducted in vineyard systems have linked repeated Cu accumulation in soil to long-term application of Cu-based fungicides [32].

Irrigation constitutes another major pathway to connect contaminated water with agricultural soils and crops. In rice-growing areas affected by industrial emissions, irrigation water was identified as a dominant Cd source under moderately contaminated conditions, whereas atmospheric deposition became the major pathway close to a smelting source. Surface runoff and leaching further contributed to the redistribution and removal of Cd within the soil–water system [33]. Wastewater irrigation may therefore increase the probability of simultaneous exposure to inorganic and organic contaminants, particularly where treated or untreated wastewater contains both trace metals and pesticide residues.

Atmospheric deposition can be especially important in peri-urban and industrially influenced agricultural areas. Source-apportionment studies have attributed substantial fractions of Pb, Zn, Cr and Cd in agricultural soils to atmospheric deposition, mining, smelting and other industrial activities, often in combination with livestock manure, fertilizer application and natural rock weathering [29,30]. These findings demonstrate that agricultural soils located near industrial or urban centers are exposed to overlapping agricultural and non-agricultural contaminant sources.

The distinction between rural and peri-urban agricultural systems is therefore important. In predominantly rural systems, contaminant inputs are more associated with fertilizer application, pesticide use, livestock manure and irrigation. By contrast, peri-urban agricultural soils may receive additional inputs from road traffic, industrial emissions, mining, wastewater and atmospheric deposition. Studies in urbanized or industrially influenced areas have reported elevated concentrations of Cd, Pb, Zn, Cu and other potentially toxic elements and have identified contributions from natural, agricultural and industrial sources [34,35].

Historical contamination also contributes to present-day co-contaminant exposure. Cross-regional assessments of soils intended for agri-food production have identified both metals/metalloids and organochlorine pesticides, with contamination attributed to combinations of geological background, historical pesticide use and mineral extraction. Such findings highlight that current agricultural exposure may reflect both contemporary inputs and legacy contamination accumulated over previous decades [36].

Pesticide residues themselves may persist in repeatedly treated agricultural soils and can become integrated into local soil microbial and transformation processes. Long-term exposure to herbicides such as linuron has been shown to select specialized pesticide-degrading microbial populations, illustrating how repeated pesticide application can establish persistent chemical and biological signatures within agricultural soils [37]. Similarly, intensive pesticide use in agricultural areas can result in persistent pesticide residues and mixed exposure conditions when metals are simultaneously present in soil or irrigation water.

Direct evidence of combined contamination has been reported for several metal–pesticide systems. Cd and penflufen co-exposure altered pesticide persistence and bioaccumulation, while experimental studies of Cd–trichlorfon and Hg–chlorpyrifos contamination demonstrate that metal and pesticide residues can occur simultaneously in agricultural water or soil systems and may subsequently enter plant-based remediation or food-production pathways [38,39,40].

#### 3.4.2. Co-Distribution and Bioaccumulation Across Soil–Plant–Food Systems

The environmental behavior of heavy metals and pesticides in agroecosystems is governed by coupled physicochemical and biological processes, including sorption–desorption, complexation, ion exchange, redox transformation, and biotransformation. Soil organic matter involved in these processes comprises solid-phase humic substances, dissolved organic matter (DOM), and low-molecular-weight organic ligands released during decomposition or root exudation. These compounds contain carboxylate, phenolic, hydroxyl, and other functional groups capable of interacting with metal ions and pesticide molecules through complexation, hydrogen bonding, electrostatic interactions, and hydrophobic partitioning [41].

Soil pH is particularly important because it controls contaminant speciation, surface charge, and the sorption behavior of both inorganic and organic pollutants. Differences in soil physicochemical properties can therefore strongly modify contaminant bioavailability and toxicity. In soybean, for example, atrazine phytotoxicity differed markedly between two soil types, with EC50 values for plant height of 23.44 mg kg^−1^ in black soil and 12.79 mg kg^−1^ in yellow-brown earth, while corresponding root-length EC50 values were 54.29 and 21.48 mg kg^−1^, respectively. Atrazine adsorption was also higher in black soil (41.56 mg kg^−1^) than in yellow-brown earth (32.95 mg kg^−1^), illustrating the importance of soil sorption capacity and DOM composition in regulating pesticide behavior [41].

The effect of pH and soil composition on pesticides is compound-specific and depends strongly on molecular structure, ionization behavior, and hydrophobicity. Consequently, contaminant mobility cannot be predicted from total organic-matter content or pH alone, but depends on contaminant speciation, pesticide properties, mineralogy, ionic strength, and the composition of organic matter. This is particularly relevant under mixed-contaminant conditions, because metals may alter pesticide sorption, root-surface interactions, and subsequent plant uptake [42].

Co-distribution patterns of metals and pesticides show considerable spatial and biological heterogeneity. Contaminants may accumulate preferentially in cultivated topsoil because this layer receives repeated pesticide applications, fertilizers, atmospheric deposition, and crop amendments, while vertical redistribution may occur through infiltration, preferential flow, colloidal transport, and irrigation. In peri-urban vegetable systems, substantial differences in metal accumulation have been reported among crop groups. The highest transfer factors for Cd, Pb and As were observed in leafy vegetables, whereas leguminous vegetables showed the highest transfer of Cu and Zn and root vegetables the highest transfer of Cr. Leafy vegetables also showed the highest estimated health risk, with a mean risk value of 1.26, and Cd was identified as the dominant risk contributor [43].

The rhizosphere represents a dynamic zone of contaminant redistribution because root exudation, microbial activity, and localized physicochemical gradients influence contaminant availability and transformation. Co-exposure to metals can modify the uptake and transport of organic contaminants; however, evidence from organophosphate esters should be interpreted with caution because these compounds are primarily industrial organophosphorus chemicals rather than agricultural pesticides. Such studies are therefore considered here only as mechanistic analogs for metal–organic contaminant interactions and are not included as direct pesticide evidence [42].

Metal-containing particles and nanoparticles may influence contaminant transport and bioavailability. In rice seedlings exposed to OPEs and CuO nanoparticles, OPE concentrations in roots and shoots were lower than in treatments containing OPEs alone or OPEs with low concentrations of Cu^2+^, which suggested that CuO nanoparticles can hinder short-term root uptake through competitive adsorption. Relatively hydrophobic OPEs accumulated in cell walls, while more hydrophilic compounds were concentrated in cell sap, which showed that physicochemical properties also affect subcellular distribution [44].

At the biological level, metal–pesticide co-exposure can alter contaminant accumulation and intensify toxicological responses. In zebrafish chronically exposed to Cd and β-cypermethrin, both contaminants accumulated in the organism, while combined exposure produced stronger oxidative stress and more pronounced histopathological damage than individual exposures. After 60 days, Cd and β-cypermethrin concentrations reached 1.21 ± 0.23 mg kg^−1^ and 0.48 ± 0.05 mg kg^−1^, respectively, and co-exposure was associated with marked tissue alterations in the brain, liver, and intestine. These findings indicate that metal–pesticide mixtures can enhance both contaminant burden and biological injury under prolonged exposure conditions [45].

Representative metal–pesticide combinations, their interaction types, and the associated environmental or biological effects are summarized in Table 6.

Quantitatively, Cd co-exposure increased the bioaccumulation of R-(−)-penflufen by up to 1.73-fold, reduced its dissipation by up to 32.3%, and increased residues by up to 5.35-fold in zebrafish [40]. In rice, Cu co-exposure enhanced the acropetal translocation of highly hydrophobic current-use pesticides and promoted the excretion of moderately hydrophobic compounds from roots, although the abstract does not report a single common percentage applicable across all 16 pesticides investigated [46]. In the Cd–trichlorfon system, Brassica juncea accumulated Cd and degraded trichlorfon, while simultaneous exposure produced a mutual inhibitory effect on contaminant removal; however, no specific percentage for the combined-removal effect is reported in the abstract [39]. For Hg–chlorpyrifos co-contaminated soil, the combination of biochar and mycorrhiza provided the strongest remediation performance among the tested amendments, but the abstract does not provide a single quantitative removal percentage that can be transferred directly into the table [46].

Plant–soil interactions also play a central role in contaminant redistribution. In tomato plants, CdSO_4_ and ZnSO_4_ were transported from soil to roots and from roots to shoots more readily than their corresponding nanoforms, CdS and ZnS quantum dots. The more rapid transport of CdSO_4_ was associated with stronger adverse effects on growth, chlorophyll content, lipid peroxidation, and antioxidant responses, showing that contaminant form can directly influence both phytotoxicity and food-chain transfer potential [47].

Metal co-exposure can alter the conventional relationship between pesticide hydrophobicity and plant translocation. In rice exposed to 16 current-use pesticides, the presence of Cu enhanced the acropetal translocation of highly hydrophobic compounds from roots to shoots and promoted the excretion of moderately hydrophobic pesticides from roots into the surrounding solution. These findings suggest that metal–pesticide co-contamination may substantially modify uptake–translocation–excretion dynamics and challenge predictions based on pesticide hydrophobicity alone [46].

The biotic components of agroecosystems further regulate contaminant fate through microbial transformation, enzymatic degradation, rhizosphere processes, and plant physiological responses. Metals and pesticides may modify each other’s persistence and toxicity by affecting microbial activity, membrane integrity, oxidative balance and xenobiotic transformation pathways. Therefore, the environmental behavior of mixed contaminants cannot be reliably inferred from single-contaminant experiments alone.

### 3.5. Mechanisms of Heavy Metal–Pesticide Co-Exposure

#### 3.5.1. Physicochemical Interaction Mechanisms

The co-occurrence of heavy metals and pesticides in agroecosystems leads to complex physicochemical interactions that can modify contaminant mobility, persistence, bioavailability, and toxicity. These interactions involve sorption–desorption processes, complexation, competition for binding sites, changes in surface partitioning, and contaminant-specific transformation processes. Importantly, the direction of these effects cannot be generalized because the same metal may increase or decrease pesticide persistence or bioavailability depending on contaminant identity, concentration, and environmental conditions [41,48].

In soils, metal ions can interact directly or indirectly with pesticide functional groups and with mineral or organic sorption surfaces. Pesticides containing carboxylate, phosphonate, amino, or other electron-donor groups may participate in metal coordination, whereas comparatively neutral compounds are influenced more strongly by hydrophobic partitioning and surface interactions. These chemical differences are important because they determine whether metal co-exposure promotes retention, competition, complex formation, or enhanced transport (Table 7).

Sorption mechanisms mainly determine the mobility and stability of co-contaminants. Clay minerals, soil organic matter and mineral surfaces can retain pesticides through adsorption, partitioning and electrostatic interactions, while the presence of metal ions may modify the properties of these sorption domains. Experimental evidence indicates that soil composition can markedly influence pesticide behavior. For atrazine, adsorption reached 41.56 mg kg^−1^ in black soil compared with 32.95 mg kg^−1^ in yellow-brown earth. Correspondingly, the EC50 for soybean root length was 54.29 mg kg^−1^ in black soil but only 21.48 mg kg^−1^ in yellow-brown earth, which demonstrated that differences in sorption and dissolved organic matter composition can substantially modify pesticide bioavailability and phytotoxicity [41].

The influence of metals on pesticide behavior is also contaminant-specific. In soil–earthworm microcosms, both Cd and Cu inhibited the dissipation of dinotefuran enantiomers, while their toxicological interactions differed: Cd–dinotefuran showed an antagonistic response, whereas Cu–dinotefuran produced a synergistic response in acute toxicity assays [42]. Metal effects also varied with concentration and altered the bioaccumulation and enantioselective behavior of dinotefuran, emphasizing that physicochemical and biological responses to co-contamination are closely interconnected [48].

Metal co-exposure can similarly modify pesticide transport within plants. In rice exposed to current-use pesticides, Cu altered root sorption, xylem and phloem translocation, and root excretion. Particularly, Cu enhanced the acropetal translocation of highly hydrophobic pesticides and promoted the excretion of moderately hydrophobic compounds from roots, challenging the conventional assumption that strongly hydrophobic organic contaminants remain preferentially restricted to roots [46].

Particle-associated transport therefore depends strongly on surface properties. Similar behavior has been observed for microplastics, whose sorption capacity for organic contaminants depends on polymer chemistry, aging, surface functional groups, and contaminant hydrophobicity [44]. Experimental studies of coexisting pesticides have demonstrated that adsorption can involve both competitive and synergistic processes and may modify contaminant bioavailability after association with plastic particles [49].

At the kinetic level, contaminant retention depends on external surface adsorption, diffusion into internal sorption domains, and desorption behavior. Soil mineralogy is particularly important. Comparative toxicity tests conducted in artificial soils containing kaolin or bentonite showed that clay type altered pesticide toxicity, an effect attributed partly to differences in interlayer structure and cation-exchange capacity. The cation-exchange capacity increased from 7.30 cmolc kg^−1^ in kaolin soil to 22.8 cmolc kg^−1^ in bentonite soil, increasing pesticide sorption and reducing bioavailability in the latter system [50].

Organic matter also plays a dual role as a sorbent and a potential carrier of contaminants. Dissolved organic matter may bind pesticides through hydrophobic and non-covalent interactions, while solid-phase organic matter can promote retention. The atrazine study described above showed that differences in DOM composition were associated with differences in pesticide adsorption and phytotoxicity, illustrating that total organic-matter concentration alone is insufficient to predict contaminant behavior [41].

Co-contamination can additionally affect oxidative and metabolic processes associated with contaminant transformation. Under combined chlorpyrifos and Cd exposure, both contaminants induced oxidative damage in *Plutella xylostella*, but their interaction was antagonistic and involved suppression of antioxidant capacity, together with disruption of energy and amino-acid metabolism. These findings demonstrate that physicochemical co-exposure can propagate into distinct metabolic responses and that combined effects should not automatically be interpreted as synergistic [51].

Interactions among metals, pesticides and additional environmental matrices can be even more complex. In Trichoderma harzianum, Zn(II) at 5 mM and Cu(II) at 2.5 mM stimulated fungal growth and enhanced metolachlor degradation, while combined exposure to metolachlor, LDPE and Cu altered antioxidant activity, lipid peroxidation and membrane composition. These findings illustrate how metal availability and additional sorptive phases such as microplastics may jointly affect pesticide degradation and microbial adaptation [52].

#### 3.5.2. Plant Cellular and Molecular Responses

Co-exposure to metals and pesticides can intensify oxidative stress through enhanced formation of reactive oxygen species (ROS), particularly superoxide radicals (O_2_•^−^) and hydrogen peroxide (H_2_O_2_). Excessive ROS accumulation promotes membrane lipid peroxidation, chlorophyll degradation, disruption of cellular redox balance, and inhibition of plant growth and photosynthetic processes. This response is clearly illustrated in tomato plants co-exposed to cadmium and imidacloprid, where both contaminants individually increased H_2_O_2_ and lipid peroxidation, while combined exposure further aggravated oxidative stress and growth inhibition, indicating a predominantly synergistic interaction. Interestingly, chlorophyll inhibition showed an antagonistic response, demonstrating that mixture effects may differ according to the biological endpoint considered [53].

Plants respond to oxidative stress through a coordinated antioxidant network. Superoxide dismutase (SOD) constitutes a primary defense by converting O_2_•^−^ into H_2_O_2_, which is subsequently removed mainly through catalase (CAT), peroxidases and the ascorbate–glutathione system. Non-enzymatic antioxidants, including glutathione, ascorbate, carotenoids and phenolic compounds, further contribute to ROS scavenging and redox buffering. The importance of this antioxidant machinery is evident under metal stress: in Cd-contaminated cotton, biochar and biofertilizer increased leaf SOD activity by 47.70% and 77.21%, respectively, and CAT activity by 35.40% and 72.82%. In roots, SOD increased by 33.62% and 39.37%, whereas CAT increased by 36.91% and 60.29%, respectively. These treatments simultaneously reduced MDA accumulation and Cd uptake, confirming the close relationship between antioxidant defense and protection from metal-induced oxidative stress [54].

Comparable oxidative responses are induced by pesticide exposure. Sulfur deficiency in imidacloprid-exposed lettuce increased pesticide accumulation and the formation of toxic metabolites while further enhancing ROS production and lipid peroxidation. At the same time, reduced thiol availability impaired xenobiotic detoxification and suppressed the activity or expression of cytochrome P450 enzymes, glutathione-S-transferases (GSTs), and glycosyltransferases. These findings demonstrate that the cellular capacity to maintain glutathione-dependent detoxification strongly influences plant sensitivity to pesticide residues [55].

At the molecular level, contaminant exposure reprograms gene expression and metabolic networks involved in redox regulation, xenobiotic detoxification, photosynthesis and primary metabolism. GSTs are particularly important because they conjugate electrophilic pesticide metabolites with glutathione, facilitating detoxification and subsequent sequestration. The associated demand for glutathione, reducing equivalents and metabolic energy can redirect cellular resources from growth toward defense. This trade-off is reflected in the frequent reduction of biomass, chlorophyll content and photosynthetic performance under contaminant stress.

Evidence from pesticide co-exposure further supports this metabolic reallocation. In tomato seedlings simultaneously exposed to lambda-cyhalothrin and difenoconazole, combined treatment caused greater lipid peroxidation than either pesticide alone, indicating synergistic oxidative damage. Exposure increased ascorbate peroxidase, peroxidase, GST, reduced glutathione, and proline, whereas silicon supplementation improved tolerance by stimulating GSH–GST-dependent pesticide detoxification and the phenylpropanoid pathway [56].

More recent metabolomic evidence indicates that combined pesticide stress can extend beyond oxidative damage to central carbon and nitrogen metabolism. In pak choi exposed to thiamethoxam in combination with pyrethroids, combined treatments produced greater metabolic disruption than individual insecticides and increased MDA accumulation by 24.6–55.5%. Co-exposure also impaired chloroplast and mitochondrial function, reduced phytohormone levels, accelerated amino-acid catabolism, and disrupted the tricarboxylic acid cycle and phenylpropanoid biosynthesis. These effects demonstrate that oxidative stress is closely connected with broader metabolic reprogramming and crop-quality deterioration [57].

Metal-containing agrochemicals may produce similarly complex cellular responses. In soybean exposed to copper hydroxide nanowires, newly formed tissues accumulated 18–60 µg Cu g^−1^, compared with approximately 10 µg g^−1^ after treatment with a commercial Cu-based fungicide. Proteomic and metabolomic analyses revealed dose-dependent modulation of photosynthesis, energy production, fatty-acid metabolism, lignin biosynthesis and carbohydrate metabolism, whereas the commercial fungicide induced a stronger oxidative-stress and amino-acid-metabolism response [58].

**Figure 6:**
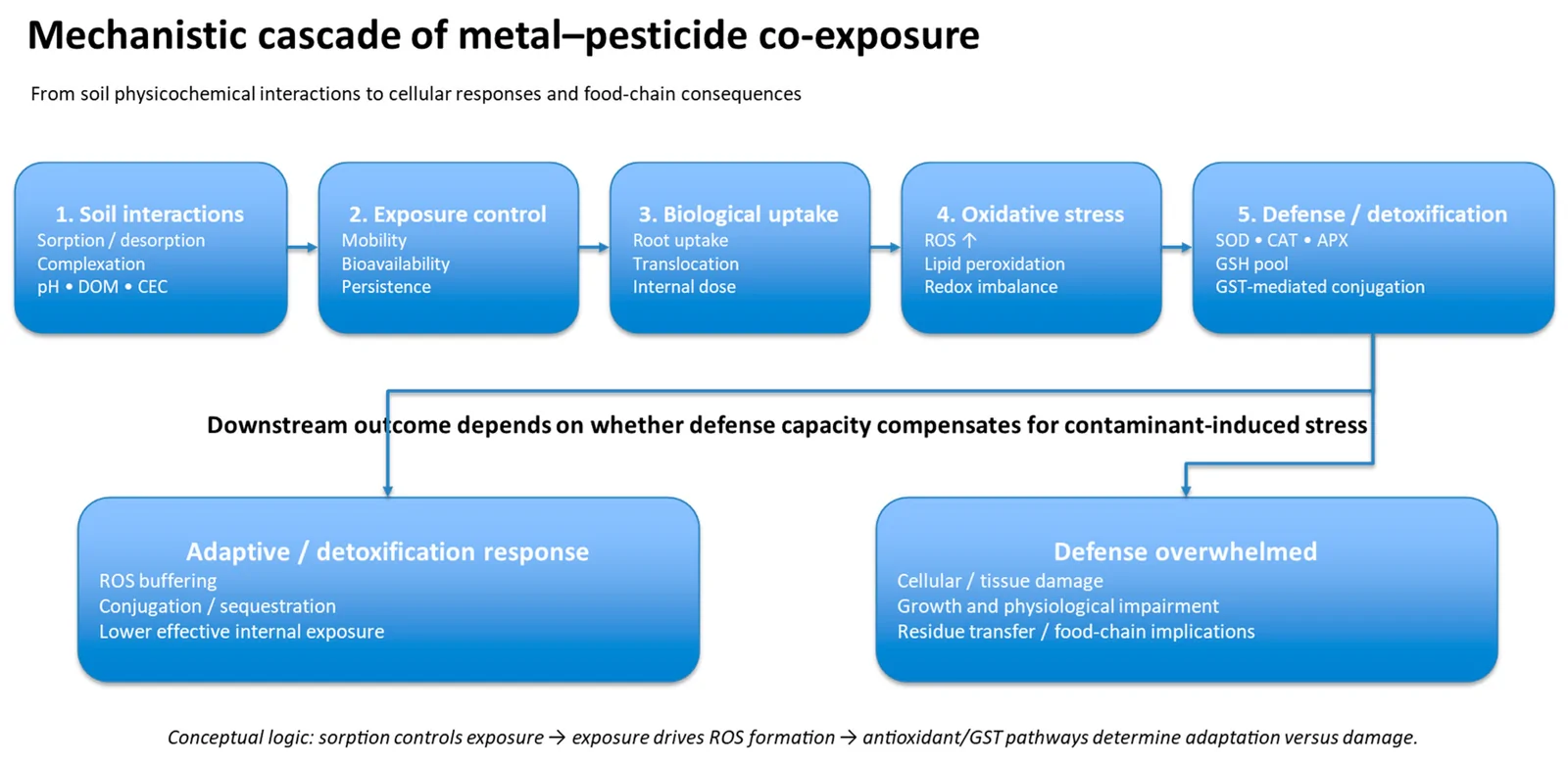
Conceptual mechanistic cascade of metal–pesticide co-exposure. Soil sorption, complexation, and related physicochemical processes regulate contaminant bioavailability and uptake, which may trigger ROS formation and antioxidant/GST-mediated responses, leading either to adaptation or to cellular damage and food-chain effects.

These findings support a mechanistic cascade in which soil-level sorption, complexation, and related physicochemical processes regulate contaminant mobility and bioavailability, thereby influencing plant uptake and internal exposure; once absorbed, co-contaminants may trigger ROS generation, antioxidant activation, and GST-mediated detoxification, with the balance between these responses determining whether plants adapt or develop cellular and physiological damage (Figure 6).

#### 3.5.3. Vertebrate and Food-Chain Toxicological Responses

In vertebrate systems, metal–pesticide co-exposure can induce oxidative stress, mitochondrial dysfunction, metabolic disturbance, inflammation, and tissue injury. These effects are commonly associated with increased ROS formation, lipid peroxidation, disruption of antioxidant defenses, and activation of apoptotic pathways. However, as observed in plants and microorganisms, the interaction between contaminants is not uniformly synergistic and may vary among species, tissues, and toxicological endpoints.

The combined effects of Cd and fipronil in the freshwater copepod *Notodiaptomus iheringi* illustrate this endpoint dependence. Both contaminants reduced survival, while fipronil also impaired reproduction. The mixture produced antagonistic responses for acute and sub-chronic survival and for egg production, whereas a synergistic interaction was observed for egg hatching. These contrasting responses demonstrate that mixture toxicity cannot be reliably inferred from the individual effects of each contaminant [59].

A similar complexity has been observed for Cd or Cu co-exposure with dinotefuran in earthworms. Cd combined with rac-dinotefuran produced an antagonistic effect on acute toxicity, whereas Cu produced a synergistic response. Both metals inhibited dissipation of dinotefuran enantiomers and altered pesticide accumulation in *Eisenia fetida*, indicating that metal co-exposure can modify both toxicological outcomes and internal contaminant burden [48].

Combined exposure can also interfere directly with oxidative-stress pathways. In microbial bioreporter systems exposed to Ag(I) or Cr(VI) together with organophosphorus pesticides, metal addition altered pesticide toxicity mechanisms, producing either synergistic or antagonistic responses. Ag(I) significantly increased pesticide-induced ROS levels, an effect associated with enhanced membrane permeability and greater cellular entry of toxic compounds [14].

In amphibians, co-exposure can elicit compensatory rather than strictly additive toxicity. Adult *Pelophylax nigromaculatus* exposed to acetamiprid and Cd showed significant metabolic and tissue injury under individual exposures, whereas combined exposure activated an adaptive compensation network that maintained redox balance and redirected amino-acid and nucleotide metabolism. This response attenuated inflammatory activation and liver injury relative to the individual treatments, illustrating that adaptive metabolic reprogramming can substantially alter the expected outcome of contaminant mixtures [60].

Pesticide toxicity in vertebrates is also closely linked to mitochondrial and oxidative damage. Difenoconazole exposure in carp increased ROS production, oxidative stress, inflammation, mitochondrial imbalance, and apoptosis in gill tissues, while antioxidant treatment reduced these effects. Similar responses have been observed in kidney and brain tissues after exposure to triazole fungicides or avermectins, supporting the central role of redox imbalance and mitochondrial dysfunction in vertebrate pesticide toxicity [61,62].

The food-chain significance of these responses lies in the fact that contaminant bioaccumulation and physiological injury may occur simultaneously. In crop systems, metals and pesticides can alter root uptake, translocation, and tissue accumulation, increasing the probability that residues enter edible plant parts. In aquatic systems, persistent pesticide residues and metal co-exposure may likewise affect organisms that occupy key trophic positions. For example, copepods such as *N. iheringi* function as important links in aquatic food webs, meaning that effects on survival and reproduction may propagate beyond the directly exposed organism [59].

Recent evidence also indicates that combined contaminant stress may impair food quality through broader metabolic disruption. In pak choi, combined insecticide exposure caused MDA accumulation of 24.6–55.5%, impaired chloroplast and mitochondrial function, and disrupted amino-acid, carbon, and nitrogen metabolism. Although this represents pesticide–pesticide rather than metal–pesticide exposure, it illustrates how mixture toxicity can translate into crop-quality deterioration and therefore into food-chain consequences [57].

## 4. Environmental and Food-Safety Implications, Mitigation Strategies, Evidence Limitations, and Future Perspectives

### 4.1. Implications for Ecosystem and Food Safety

The co-occurrence of heavy metals and pesticides in agricultural soils exerts complex, multilevel effects on soil biogeochemistry, plant physiology, microbial community dynamics, and contaminant transfer through food systems. These interactions may modify contaminant mobility, persistence, bioavailability, and biological uptake, although their magnitude and direction depend strongly on contaminant identity, concentration, soil properties, crop species, exposure duration, and environmental conditions. From a food-safety perspective, the principal concern is therefore not merely the simultaneous presence of metals and pesticides in agricultural soils, but whether their coexistence alters the fraction available for crop uptake, accumulation in edible tissues, and subsequent dietary exposure.

Deterministic HQ and HI estimates provide useful screening-level information but may not adequately represent heterogeneity in contaminant concentrations, food-consumption patterns, body weight, and other exposure variables. This limitation is consistent with the uncertainty observed in the present evidence base: among the 788 publications subjected to extended eligibility audit, only 134 (17.0%) met the eligibility criteria directly, whereas 400 (50.8%) required full-text verification because co-contamination could not be confirmed from the abstract and 254 (32.2%) were excluded. Such uncertainty in evidence classification parallels the broader need to distinguish variability from uncertainty in quantitative risk assessment. Probabilistic Monte Carlo approaches can characterize risk distributions and upper-tail exposure, sensitivity analysis can identify the variables that most strongly influence predicted risk, and two-dimensional Monte Carlo simulation can explicitly separate population variability from parameter uncertainty [63].

**Figure 7:**
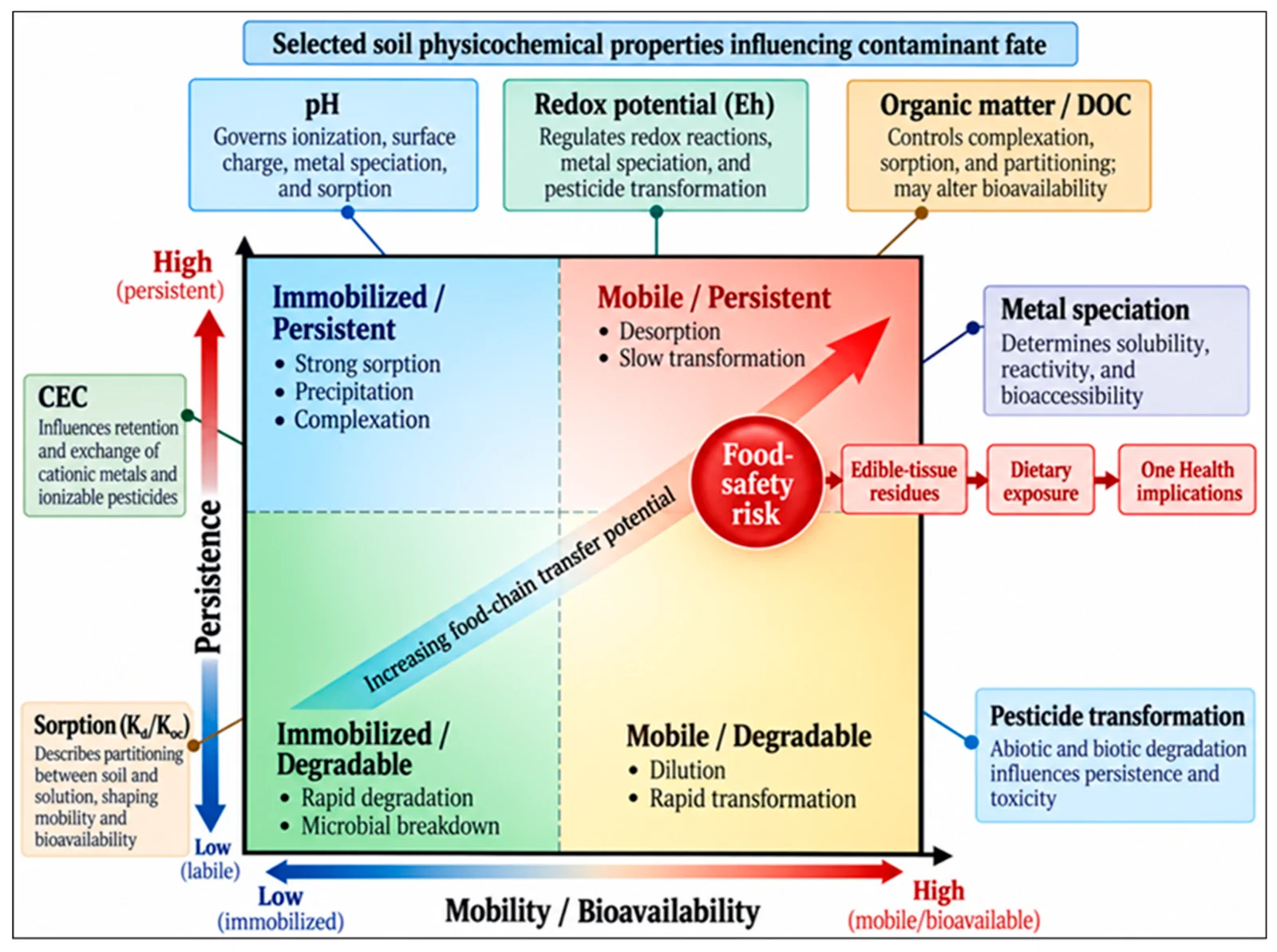
Conceptual risk matrix linking soil physicochemical properties, contaminant mobility and persistence, and soil-to-food transfer potential.

Soil physicochemical properties play a central role in this process. Soil pH affects ionization, surface charge, and metal speciation; redox potential controls metal transformation and pesticide degradation; organic matter and dissolved organic carbon influence complexation, sorption, and partitioning; and cation-exchange capacity governs the retention of cationic metals and ionizable pesticides. These processes determine whether contaminants remain immobilized, become mobile and persistent, or are degraded before reaching the food chain. The conceptual relationship between mobility, persistence, soil properties, and food-chain transfer is summarized in Figure 7.

The relevance of total soil contamination to food safety is therefore strongly conditioned by bioavailability. In intensive agricultural soils affected by long-term Cu-based fungicide use, soil Cu concentrations reached up to 217 mg kg^−1^ without producing an estimated dietary HQ >1 in the analyzed crops, whereas concentrations as high as 1700 mg kg^−1^ exerted clear selective pressure on soil microbial communities. This contrast illustrates that ecological effects and dietary risk may diverge even within the same production system [64].

Rice-based systems provide stronger evidence of direct transfer into edible tissues. In northwestern Hubei, Cd exceeded agricultural-soil regulatory limits in 89.1% of samples, while Ni and Cd exceeded food limits in 52.2% and 58.7% of rice samples, respectively. The bioavailable Cd fraction represented approximately 81.9% of total Cd, and Cd together with As accounted for 80.8% of the total non-carcinogenic risk [65]. This demonstrates that bioavailable fractions and crop-specific transfer are more informative for food-safety assessment than bulk soil concentrations alone.

Direct co-exposure experiments further show that contaminant interactions can modify accumulation in edible tissues. In lettuce simultaneously exposed to ZnO nanoparticles and imidacloprid, co-exposure increased both Zn and pesticide accumulation in shoots and aggravated biomass loss, oxidative stress, and metabolic disruption. ZnO nanoparticles enhanced conversion of imidacloprid to 5-OH-imidacloprid by 5.8–130%, depending on particle size and pesticide concentration. Larger particles favored higher Zn and imidacloprid accumulation in shoots, whereas smaller particles promoted pesticide metabolism more efficiently [66].

Pesticide stereochemistry may also affect metal transfer. Napropamide enantiomers produced distinct effects on heavy-metal accumulation: S-napropamide reduced plant biomass and metal accumulation, while the more herbicidally active R-enantiomer increased root biomass and metal accumulation through regulation of uptake and transport genes. Path coefficients linking biomass, gene regulation, and heavy-metal content reached 0.235 and 0.683, respectively [67]. These results indicate that co-contamination risk may depend not only on contaminant identity but also on molecular form.

Plants growing in contaminated soils may simultaneously experience oxidative, nutritional, and metabolic stress. Metals can interfere with the uptake of essential elements, whereas pesticides may disrupt photosynthesis, redox balance, and primary metabolism. These physiological effects become especially relevant when they modify translocation to edible organs. In vegetable systems from Bangladesh, soil-to-plant transfer followed the trend Cd > Pb > Cr, although THQ, total THQ, and HI remained below 1 under the studied exposure conditions [68].

A stronger food-chain signal was observed in the Yamuna floodplain, where 84 sediment and 56 vegetable samples were evaluated for 20 organochlorine pesticides and nine metals. Translocation factors >1 were common for Ni, Cr, Cu, and Zn, while several OCPs also showed high transfer into vegetables. Spinach exhibited the highest metal contamination, HQ exceeded 1 for radish and cauliflower consumption in children, and HI exceeded 1 for nearly all vegetables except onion leaves [69].

Because food-safety datasets now span multiple commodities and exposure scenarios, the most relevant quantitative findings are summarized in Table 8.

Dietary-risk studies show substantial variability among commodities and exposed populations. Large surveillance datasets may indicate relatively low average population risk despite occasional MRL exceedances, whereas probabilistic analyses can reveal considerably higher exposure in vulnerable subgroups. In China, analysis of 6554 fruit and vegetable samples detected highly toxic pesticides in 4.96% of samples, with 1.57% above national MRLs, yet mean mixture HI values remained <1 for adults and children [70]. In contrast, probabilistic assessment of pistachios produced a 95th-percentile total hazard quotient of 1.471 in adults and 10.644 in children, emphasizing the influence of consumption patterns and body weight [72].

Rice is particularly important because of its high consumption rate and ability to accumulate multiple contaminant classes. In one multi-contaminant assessment, HI values were 2.10 for Pakistani rice, 1.86 for Indian rice, 1.01 for Iranian rice, and 0.98 for Thai rice. Arsenic dominated risk in Iranian and Thai rice, whereas diazinon contributed more strongly in Pakistani and Indian rice [73]. A more recent Iranian study integrating Pb, Cd, Cr, Ni, diazinon, and mycotoxins found HI > 1 in all region × season combinations, with maxima of approximately 5.86 in adults and 11.94 in children. Cr and ochratoxin A were the principal risk drivers, whereas diazinon contributed only minimally [74].

The importance of accounting for consumer-group variability is further illustrated by recent probabilistic dietary-exposure assessments. In a study of 197 pepper samples, pesticide residues were detected in 27% of samples and multiple residues in 14%, with concentrations ranging from 0.02 to 0.94 mg kg^−1^. Chronic dietary exposure remained below the health-risk threshold across all evaluated consumer groups, whereas acute risk exceeded the acceptable threshold in some scenarios, particularly for children. Sensitivity analysis identified body weight and ingestion rate as the dominant parameters influencing exposure estimates, demonstrating how consumer characteristics can substantially affect upper-tail dietary-risk estimates [83].

Animal-derived foods provide another pathway of trophic transfer. In Romanian dairy systems, Pb and DDD exceeded MRLs in milk and cheese from all three investigated areas, while Cu and Zn exceeded limits in cheese from two areas. Source-specific HQ values >10 for Pb and >1 for Cu and total DDT indicated potentially relevant dietary exposure [75].

Fish and seafood similarly integrate contaminants accumulated through aquatic food webs. Some studies report individual THQ values below 1 despite measurable contamination, whereas cumulative indices exceed acceptable thresholds. In dried fish from Bangladesh, pesticide-risk indices were low, but carcinogenic risk associated with metals remained more relevant [76]. In an impacted Indian estuary, mean HI exceeded 1 for children despite generally acceptable individual THQ values [78]. In Sphoeroides spp., multiple metals and OCPs were simultaneously detected, and THQ exceeded 1 at a consumption rate of 120 g day^−1^ [77].

Direct co-exposure studies provide mechanistic support for these field observations. In *Labeo rohita*, combined Pb and endosulfan exposure increased tissue accumulation by up to 1.7-fold, damaged erythrocytes exceeded 65%, and the genetic damage index increased approximately threefold in the combined high-dose group. Significant Pb × endosulfan interactions confirmed synergistic toxicity, demonstrating that concurrent metal–pesticide exposure may alter both internal dose and biological response [82].

Food processing can partly modify dietary exposure after contaminants enter the food chain. In shrimp, pesticide residues ranged from 0.007 to 0.703 µg kg^−1^ in raw tissue, while frying and microwave cooking reduced residues by 59.4% and 60.3%, respectively. Boiling and grilling were less effective, with reductions of 48.8% and 51.3% [80]. These effects are pesticide-specific and should not be extrapolated to metals.

Pesticide residues in bee products further illustrate the connection between ecosystem health and food safety. Residues were detected in 25.4% of beebread samples and 2.8% of honey samples, with low estimated risk for human consumers but continued concern for colony-level effects, particularly for thiamethoxam [81].

Food-safety implications also extend beyond dietary exposure alone. In intensely cultivated areas, occupational contact with pesticides may contribute substantially to total exposure. Among Indian farmworkers not using protective equipment, pesticide-related HQ values ranged from 0.02 to 1029.82, with HI > 1, while PPE use significantly reduced both residue concentrations and the number of detected pesticides [84]. Population-based studies from intensive agricultural areas have also reported associations between environmental pesticide/heavy-metal exposure and chronic kidney disease, supporting the need for integrated environmental and biomonitoring approaches [85].

At broader spatial scales, risk is strongly source- and age-dependent. In the Lower Yangtze River Basin, mean total carcinogenic risk from metals reached 1.09 × 10^−4^ in children and 2.35 × 10^−5^ in adults, while corresponding organic-pollutant risks were 1.38 × 10^−5^ and 5.03 × 10^−6^. Oral ingestion represented the dominant exposure pathway, and children were consistently the most sensitive receptors [86].

Spatially explicit monitoring reinforces this age-related vulnerability. In agricultural soils and vegetables from southeastern Shiraz, the Hazard Index reached 1.55 in children and 0.22 in adults, while the maximum health-risk index reached 20.42 for Ni in children. Ingestion contributed more strongly to total risk than inhalation or dermal exposure [87].

The food-safety relevance of contaminants is not restricted to conventional crops and livestock. Seaweeds, increasingly used as food and feed resources, can accumulate metals, persistent organic pollutants, and pesticides. In New Caledonian species, all analyzed samples exceeded the European total-arsenic maximum used for food additives, approximately half exceeded French recommendations for inorganic arsenic in seaweed products, and only five species were considered suitable for consumption under the applied framework [88].

Because residues occur at low concentrations within complex food matrices, analytical surveillance remains critical. Emerging sensing platforms can support rapid screening and field-based monitoring. A porphyrin MOF nanosystem achieved a triazophos detection limit of 0.6 ng mL^−1^ and approximately 85% photocatalytic degradation [89]. Nanocellulose-based SERS substrates detected organophosphorus and methyl parathion residues on apple peel at 10^−7^ g L^−1^ [90]. Electrochemical platforms have achieved detection limits of 1.67 nM for benomyl, 2.1 nM for methyl parathion, and 0.05 µg mL^−1^ for cypermethrin in food or environmental matrices [91,92,93].

Portable detection is also increasingly feasible. Smartphone-assisted MOF-based membranes have been developed for visual and on-site quantification of pesticide residues in real food samples [94], while dual-signal fluorescence/phosphorescence systems enable rapid thiram detection in fruit and vegetable matrices [95]. These technologies complement conventional chromatography and mass spectrometry and may improve routine surveillance where rapid decision-making is required.

### 4.2. Climate Modulation of Co-Contaminant Fate and Food-Chain Transfer

Climate change is increasingly recognized as an active driver of contaminant behavior rather than a background environmental variable. Changes in temperature, precipitation, soil moisture, evapotranspiration, flooding frequency, drought intensity, and atmospheric CO_2_ can modify contaminant sorption, degradation, transport, biological uptake, and trophic transfer. These effects are especially important in agroecosystems already affected by legacy heavy metals or pesticide residues, where climate-related changes can alter both exposure pathways and contaminant persistence.

The influence of climate on contaminant fate is clearly illustrated by modeling studies of pesticide transport. Future-climate simulations for atrazine and estrone in agricultural soils predicted higher air temperatures and evapotranspiration, leading to drier soil conditions and reduced biodegradation in the aqueous phase. At the same time, enhanced plant transpiration increased root uptake, while infiltration and leaching were projected to decline. Thus, warmer and drier conditions may simultaneously reduce contaminant transport to groundwater while increasing retention or plant-associated uptake within the soil profile [96].

Temperature can also modify pesticide dissipation and ecological response. In outdoor freshwater mesocosms exposed to 1 µg L^−1^ imidacloprid, warming by +4 °C and simulated heatwaves of up to +8 °C shortened the average pesticide dissipation half-life from approximately 4 days under ambient conditions to 3 days under both warming scenarios. Despite faster dissipation, community recovery from imidacloprid stress was slower during heatwaves, indicating that lower chemical persistence does not necessarily translate into lower ecological toxicity. Zooplankton communities were particularly sensitive, and structural-equation modeling revealed stronger stressor effects at higher trophic levels [97].

The timing and frequency of contaminant inputs may be as important as temperature itself. In shallow-lake microcosms, agricultural runoff containing three pesticides—terbuthylazine, tebuconazole, and pirimicarb—together with Cu and nitrate was tested at 22 and 26 °C. Multiple-pulse exposure caused stronger effects on the grazing snail *Lymnaea stagnalis* and periphyton than the same total contaminant load delivered as a single dose. Warming had only limited direct effects in this experiment, but indirect trophic interactions substantially altered ecological responses, showing that climate–contaminant interactions may emerge through food-web structure rather than only through direct toxicological effects [98].

Temperature may additionally alter sorption and remobilization of pesticides associated with particulate carriers. In aged polyethylene microplastics, terbuthylazine sorption declined relative to pristine particles because weathering introduced oxygen-containing functional groups and reduced surface hydrophobicity. Sorption also became less favorable at higher temperatures, while thermodynamic analysis suggested that warming could promote re-release of previously sorbed pesticide into the environment. These findings indicate that climate warming may influence not only pesticide degradation but also secondary release from aged environmental particles [99].

Floodplain systems illustrate the importance of such hydrological fluctuations. Alternating flooding and drought produce large changes in water-table height, oxygen availability, and microbial metabolism. Metagenomic analysis of floodplain sediments showed that nitrifying communities possess broad genetic potential for utilizing organic nitrogen compounds, including urea, cyanate, nitriles, and related substrates. These microbial functions are relevant because hydrological oscillations influence both nutrient cycling and the transformation of agricultural contaminants, including compounds derived from fertilizers and pesticides [100].

Climate-related hydrological stress can also influence remediation capacity. In multi-metal systems, *Bacillus* sp. SSAU-2 retained substantial Cr(VI)-removal capacity in the presence of several metals, although Hg and Cd strongly inhibited its performance. The strain normally achieved approximately 83% Cr(VI) removal, whereas only 0.1 ppm Hg(II) reduced removal efficiency to 32%. Zn(II), Fe(III), and Cu(II), in contrast, stimulated growth and Cr(VI) removal. Although this study focused on multi-metal rather than metal–pesticide contamination, it illustrates how changing pollutant mixtures can strongly influence microbial remediation performance under environmentally variable conditions [101].

Climate–contaminant interactions can also alter biological transfer across trophic levels. Vineyard agroecosystems provide a particularly relevant example because Cu-based fungicides create long-term metal accumulation while increasing temperatures modify insect development and biological-control interactions. Under combined warming and Cu exposure, survival and development of the grapevine moth *Lobesia botrana* and its parasitoid *Trichogramma oleae* were altered in stage-specific ways. Warmer conditions accelerated moth development, whereas increasing Cu exposure delayed development and reduced pupal mass. In the parasitoid, emergence decreased and development time increased under combined high-temperature and Cu exposure, while size, longevity, and fecundity declined at high Cu levels [102].

A subsequent study directly evaluated Cu accumulation under current and projected warmer conditions. Increasing environmental Cu concentrations raised Cu accumulation in *L. botrana*, while tissue concentrations declined across developmental stages, indicating internal regulation. Importantly, high Cu exposure combined with warming increased Cu accumulation, while warming accelerated development and increased larval mass. These results suggest that climate warming can simultaneously improve insect performance and enhance contaminant transfer through trophic pathways, potentially modifying exposure of predators and parasitoids [103].

Plant responses are similarly influenced by interactions between climate stress and contaminant exposure. Drought, salinity, heat, metals, and pesticides frequently converge on oxidative stress, altered membrane stability, and changes in antioxidant metabolism. Under water deficit, ZnO nanoparticle seed priming has been shown to partially compensate for drought-induced damage in rice. Drought alone reduced plant height by 31%, fresh weight by 22%, and dry weight by 28%. Priming with 25 ppm ZnO nanoparticles reduced MDA by approximately 53% and increased SOD, CAT, and POD activities by 11%, 13%, and 38%, respectively, while improving yield-related traits [104].

Microbial symbionts may provide another route to climate resilience. The multi-stress-tolerant bacterium Beijerinckia fluminensis BFC-33 tolerated drought, salinity, temperature stress, metals, and fungicides while maintaining plant-growth-promoting activity. Inoculated wheat exposed to different stresses showed root lengths ranging from approximately 17–24.2 cm depending on stressor combination, and the strain also displayed strong antagonistic activity against Alternaria alternata (79.2%) and Rhizoctonia solani (69%). Such multi-stress-tolerant microorganisms may therefore support crop performance where climate stress and chemical contamination occur simultaneously [105].

Broader plant-growth-promoting bacterial strategies are also increasingly discussed as climate-adaptive tools. Plant-associated bacteria can enhance tolerance to drought, salinity, heat, cold, and waterlogging while simultaneously improving nutrient acquisition and reducing dependence on chemical inputs. Their relevance to co-contamination lies in their ability to maintain plant growth and microbial functionality under environmental conditions that may otherwise increase contaminant stress or reduce natural attenuation [106].

Climate adaptation can also involve reducing agrochemical use itself. Chitosan–copper combinations showed synergistic antifungal activity that allowed a 50% reduction in Cu concentration while maintaining antifungal efficacy. Because Cu-based fungicides are a major source of long-term Cu accumulation in some agricultural soils, such formulation strategies may reduce future contaminant loading while preserving crop protection [24].

Other emerging approaches aim to increase stress tolerance without increasing chemical pesticide inputs. Complementary peptides designed to regulate plant proteins have been shown to modify growth, pathogen resistance, and heat-stress tolerance and may represent a future route for reducing reliance on conventional agrochemicals under warming conditions [107].

Climate change may also indirectly increase chemical-exposure pressure through changes in pest distribution and pesticide demand. Evidence from human biomonitoring in agricultural regions illustrates the downstream relevance of such trends. In Haryana, India, DDT and DDE were detected in 4% and 5% of breast milk samples, respectively, with maximum concentrations of approximately 28 and 107 µg L^−1^. Although the measured levels were considered low and did not indicate infant risk in that pilot study, the findings demonstrate how persistent agricultural contaminants can reach human tissues and support the need for longitudinal monitoring as pesticide use patterns respond to climate and food-production pressures [108].

Climate-related changes may also interact with natural food toxicants. In vitro studies of ciguatoxins and pyrethroid pesticides demonstrated strong synergistic effects on voltage-gated sodium channels, with combined exposure producing greater disruption than either contaminant alone. Although this represents pesticide–biotoxin rather than metal–pesticide co-exposure, it is relevant to climate-sensitive food safety because warming and altered marine distributions can change the geographical occurrence of biotoxins while agricultural pesticide exposure continues simultaneously [109].

**Figure 8:**
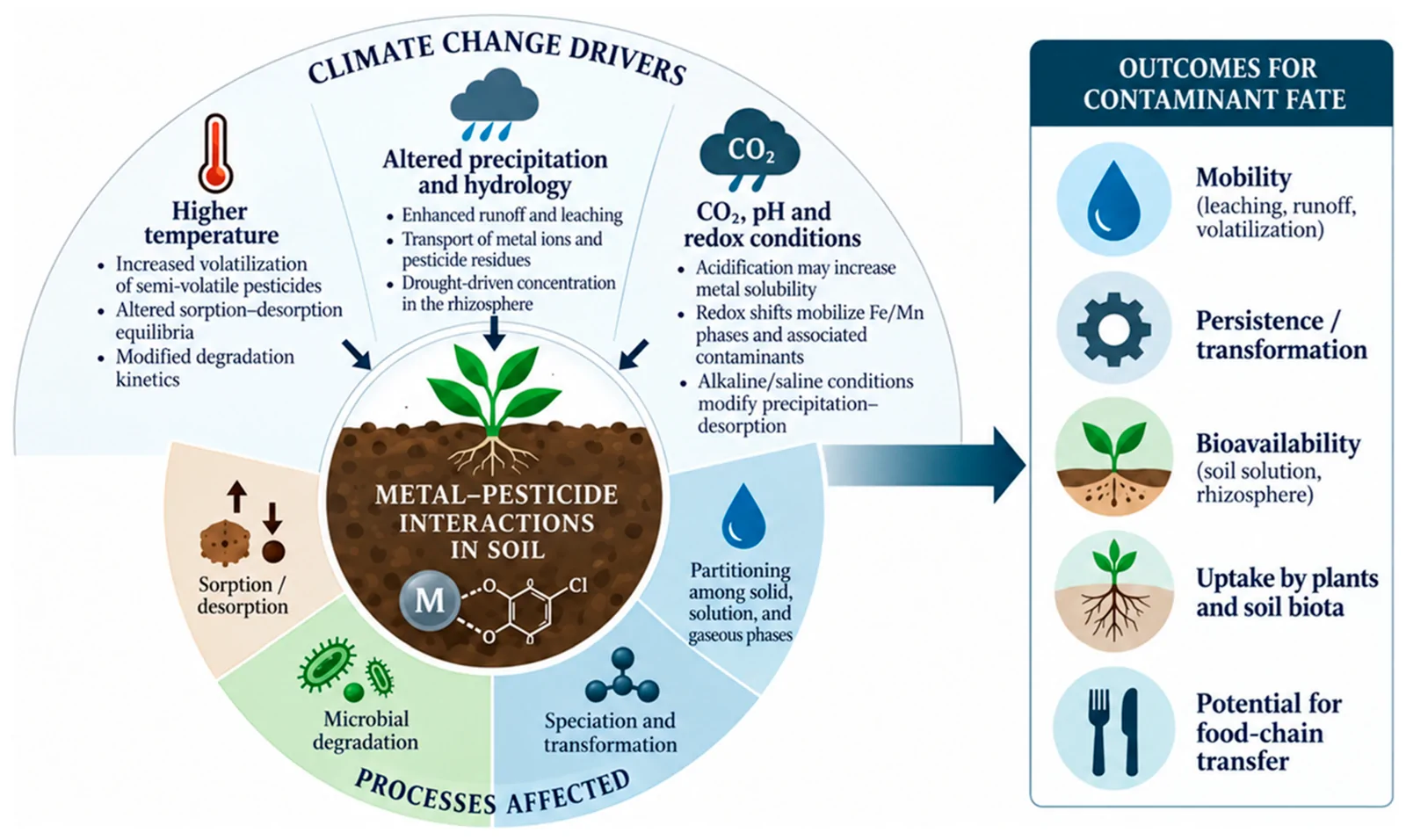
Climate-driven modulation of metal–pesticide fate and food-chain transfer.

Collectively, these mechanisms can be conceptualized as a climate-driven cascade in which temperature, precipitation, drought, flooding, soil moisture, hydrology, and biological stress modify contaminant sorption, degradation, microbial transformation, biological uptake, and trophic transfer. These interactions ultimately determine whether contaminants remain immobilized, are degraded, or become more available for plant or animal uptake, plant uptake, and potential transfer through the food chain (Figure 8).

At the ecosystem level, climate–contaminant interactions are strongly context- and endpoint-dependent. Warming shortened imidacloprid dissipation from approximately 4 to 3 days, yet heatwaves delayed biological recovery and enhanced community-level effects, demonstrating that faster chemical dissipation does not necessarily imply lower ecological risk [97]. Likewise, higher temperatures reduced terbuthylazine sorption to aged polyethylene microplastics and may favor pesticide re-release, whereas warming increased Cu accumulation in *Lobesia botrana* under high-Cu exposure [99,103].

Hydrological and physiological responses may act in opposite directions. Future-climate simulations for atrazine predicted reduced biodegradation under drier soils, but also lower infiltration and leaching and greater plant uptake associated with increased transpiration [96]. These contrasting effects indicate that climate change may redistribute exposure rather than simply increase or decrease contaminant persistence.

Climate-adaptive management should, therefore, combine contaminant control with crop and microbial resilience. Multi-stress-tolerant plant-growth-promoting bacteria can support plant performance under simultaneous drought, salinity, metal, and pesticide stress [105,106], while chitosan–Cu formulations have demonstrated that Cu input can be reduced by 50% without loss of antifungal efficacy [24]. Such strategies may help reduce future contaminant loading while maintaining crop protection under increasingly variable climatic conditions.

### 4.3. Sustainable Mitigation Strategies

Remediating agricultural soils co-contaminated with heavy metals and pesticides requires strategies capable of reducing contaminant mobility and toxicity while preserving soil fertility, microbial functionality, and crop productivity. Conventional physicochemical approaches, including excavation, soil washing, chemical immobilization, and electrokinetic treatments, may effectively remove or stabilize contaminants, but their intensive use can be constrained by cost, energy demand, disturbance of soil structure, and limited suitability for diffuse agricultural contamination. Consequently, increasing attention has shifted toward eco-remediation approaches that combine biological, agronomic, and physicochemical mechanisms to reduce contaminant bioavailability while supporting restoration of soil functions.

Phytoremediation remains one of the most accessible biological approaches for metal-contaminated agricultural soils. Its efficiency depends strongly on plant species, contaminant availability, biomass production, and the capacity to tolerate oxidative and physiological stress. In vineyard soils affected by long-term Cu-based fungicide application, spontaneous plant species showed preferential Cu accumulation in roots, and *Lolium multiflorum*, *Cyperus compressus*, and *Chrysanthemum leucanthemum* displayed phytostabilization potential during winter. Among the tested species, *L. multiflorum* combined relatively high biomass production with Cu accumulation and was considered particularly useful for decreasing Cu availability in soil [32]. Similarly, Cu-tolerant grasses such as *Paspalum notatum* and *P. urvillei* showed physiological traits favorable for phytoremediation, including higher Cu translocation, increased antioxidant-enzyme activity, larger root systems, and greater release of soluble organic carbon capable of complexing Cu^2+^ in the rhizosphere [110] (Table 9).

The effectiveness of phytoremediation can be substantially enhanced through rhizosphere-assisted processes. Plant root exudates provide carbon substrates, signaling compounds, and low-molecular-weight organic acids that stimulate contaminant-degrading microorganisms and improve rhizosphere colonization. In maize rhizosphere soil contaminated with bensulfuron-methyl, the degrading bacterium *Hansschlegelia zhihuaiae* S113 achieved 80.9% degradation, while addition of *L*-malic acid, a naturally occurring component of maize root exudates, resulted in complete pesticide degradation. The organic acid enhanced bacterial chemotaxis and root colonization, producing higher remediation efficiency than fumaric acid (89.7%) or tartaric acid (87.0%) supplementation [111]. These findings demonstrate that remediation can be improved not only by introducing degrading microorganisms but also by manipulating the chemical environment that supports their establishment and activity.

Microbial bioaugmentation represents another major component of sustainable remediation. Pesticide-degrading bacteria can transform persistent xenobiotics while simultaneously colonizing plant tissues and rhizosphere compartments. *Pseudomonas* sp. F1 removed 92.1% of epoxiconazole within six days and reduced its soil half-life from 96.3 days in uninoculated soil to 2.64 days following inoculation. Colonization of roots, stems, and leaves was associated with reduced pesticide accumulation and translocation in vegetables, indicating that microbial remediation may contribute simultaneously to soil decontamination and safer crop production [112].

Microbial consortia may offer greater functional stability than individual strains, particularly where multiple pesticides coexist. A bacterial consortium isolated from agricultural soil tolerated monocrotophos concentrations up to 2000 ppm, while individual strains showed tolerance between 500 and 5000 ppm. After two weeks, the consortium completely degraded monocrotophos in contaminated soil, with organophosphate-degrading genes detected in several constituent strains [124]. More recently, rationally assembled hexa-bacterial consortia containing biosurfactant-producing and plant-growth-promoting bacteria almost completely degraded chlorpyrifos and cypermethrin and removed up to 93.6% of lindane within eight days. In pesticide-amended soil, the best-performing consortium completely degraded chlorpyrifos and cypermethrin and removed 98.1% of lindane within 28 days. Biosurfactant production reduced surface tension from 55.8 to 30.3–32.2 mN m^−1^, facilitating pesticide solubilization and biodegradation [117].

Combined biological processes may further improve remediation efficiency. In wastewater contaminated with pentachlorophenol, bioaugmentation combined with adsorption removed approximately 97% of the pesticide within 48 h, whereas bioaugmentation combined with phytoremediation achieved approximately 90% removal after 72 h. Importantly, treated wastewater supported 95% alfalfa seed germination, whereas untreated PCP-contaminated wastewater completely inhibited germination [113]. These results illustrate the potential benefit of combining biological degradation with sorptive or plant-based processes rather than relying on a single remediation mechanism.

Organic amendments, particularly biochar, provide an additional means of controlling mixed contamination. Their porous structure, surface functional groups, mineral components, and cation-exchange capacity allow simultaneous sorption or stabilization of inorganic and organic contaminants. Biochar may also improve soil structure, nutrient retention, and microbial habitat. These functions are particularly important in metal–pesticide mixtures, where an amendment should ideally reduce metal bioavailability while also limiting pesticide mobility without suppressing microbial degradation.

A strong example is the remediation of soils co-contaminated with Hg and chlorpyrifos using *Phaseolus vulgaris* combined with biochar, mycorrhizae, and compost. All tested amendments enhanced remediation, but the combination of biochar and mycorrhiza showed the best overall performance for simultaneous Hg and chlorpyrifos remediation [38]. This type of integrated plant–amendment–microbial approach is especially relevant to agricultural co-contamination because the two contaminant classes require fundamentally different removal mechanisms: metals must be immobilized, sequestered, or extracted, whereas pesticides can additionally undergo biological or chemical degradation.

Hybrid biochar materials can provide even stronger stabilization. A Zn-modified biochar-supported Mg–Fe layered double hydroxide developed for Cd–atrazine co-contaminated soil achieved maximum adsorption capacities of 268.45 mg g^−1^ for Cd(II) and 165.04 mg g^−1^ for atrazine. After 28 days, atrazine and Cd concentrations in leachates decreased by 89.7% and 90.3%, respectively, while cumulative leaching losses declined by 44.55% for atrazine and 57.70% for Cd. The amendment also increased the non-desorbable atrazine fraction to 65.03% and the stable Cd fraction to 49.34%. In maize, atrazine bioconcentration and translocation factors decreased by 63.52% and 59.27%, respectively, whereas the corresponding reductions for Cd were 71.71% and 32.48% [116]. This study provides particularly strong evidence that engineered amendments can simultaneously control the environmental behavior of a metal and a pesticide in the same soil system.

Biopolymer-based materials provide another emerging approach. Porous microspheres produced from sodium alginate and carboxymethyl cellulose improved physicochemical and biological properties of Cu- and hymexazol-contaminated rice soil. The treatment increased nitrate nitrogen by 7.19–41.79%, available phosphorus by 4.42–24.58%, and soil urease and sucrase activities by 6.74–27.10%. At the same time, Cu and hymexazol accumulation in rice roots declined by 18.46% and 18.67%, respectively, while beneficial fungal communities were enriched [115]. Such systems illustrate a transition from contaminant immobilization alone toward multifunctional materials capable of combining remediation with biological restoration of the soil.

Microbial siderophores and other biologically produced chelators can also contribute to metal remediation while simultaneously supporting disease control. *Bacillus amyloliquefaciens* BM3 displayed high resistance to As(V) and As(III), with minimum inhibitory concentrations of 475 and 24 mM, respectively, and reduced As(V) and As(III) concentrations by 74% and 88.1%. The strain also showed broad antifungal activity and reduced *Fusarium* wilt in brinjal, suggesting that microbial remediation can be coupled with biological crop protection [114]. Microbial biosurfactants provide a complementary mechanism by forming metal–biosurfactant complexes and increasing solubilization of hydrophobic organic contaminants, thereby facilitating contaminant mobilization and microbial degradation [125].

Sustainable mitigation should not be restricted to remediation after contamination has occurred. Reducing dependence on conventional pesticides and introducing lower-impact crop-protection technologies can prevent further accumulation of organic contaminants and, in some cases, decrease reliance on Cu-based fungicides. Several nano-enabled and biologically derived alternatives from the prioritized evidence corpus illustrate this preventive approach.

Silica-coated porous ZnO nanoparticles have been proposed as slow-release fungicide carriers. The silica coating reduced the phytotoxicity associated with ZnO nanoparticles while maintaining antifungal activity, allowing the carrier and pesticide cargo to contribute jointly to crop protection [126]. Similarly, a corn-stover-templated ZnO–TiO_2_ nanomaterial achieved 79.83% control of bacterial leaf spot disease in tomato while promoting plant growth and photosynthesis [118]. ZIF-8 nanoparticles also demonstrated strong activity against *Xanthomonas oryzae* pv. *oryzicola*, with an EC50 of 0.15 mg mL^−1^, while simultaneously stimulating antioxidant defenses in rice [123].

Myco-synthesized CuO nanoparticles offer another example of a dual-function crop-protection technology. The particles inhibited *Fusarium falciforme* mycelial growth by 33.95%, and a concentration of 200 mg L^−1^ was selected as an effective treatment without detectable phytotoxicity. In greenhouse experiments, the treatment restored photosynthetic pigments, improved antioxidant balance, and increased potato tuber yield by up to 40% in healthy plants, while the commercial fungicide showed phytotoxic effects on tuber initiation [119]. Although Cu-based nanomaterials still require careful evaluation of environmental persistence and accumulation, such studies indicate that formulation and delivery strongly influence both efficacy and phytotoxicity.

Botanical compounds and plant-derived elicitors provide a second route toward reducing synthetic pesticide use. Genistein displayed an EC50 of 6.53 µg mL^−1^ against *Alternaria solani*, compared with 8.35 µg mL^−1^ for chlorothalonil, and achieved 48.1% protective and 42.5% therapeutic efficacy in tomato. Its antifungal action involved disruption of energy metabolism, ROS accumulation, membrane lipid peroxidation, and apoptotic-like responses [120]. Physcion showed an EC50 of 3.30 mg L^−1^ against *Penicillium choerospondiatis*, acting through membrane disruption, increased permeability, altered ergosterol metabolism, and excessive ROS production [121]. An experimental aldehyde–thiourea/thiazolyl hydrazine derivative, LN18, showed even stronger activity against *Monilinia fructicola*, with an EC50 of 0.17 µg mL^−1^, again involving membrane damage and ROS accumulation [122].

Plant priming provides another preventive strategy. Oregano essential-oil vapor applied to grapevine modified phenylpropanoid metabolism, activated ROS-related defense signaling, and promoted accumulation of terpenes and other potential phytoalexins associated with resistance to downy mildew. Such approaches may reduce reliance on synthetic and Cu-based pesticides by enhancing endogenous plant defense rather than directly increasing chemical inputs [127].

Biological control using beneficial microorganisms can similarly contribute to pesticide reduction while improving crop resilience. Endophytic bacteria and Zn nanoparticles have been used to address the combined challenge of heavy-metal toxicity and *Rhizoctonia solani* infection in tomato [128,129]. Treatments reduced disease severity, enhanced biomass and photosynthetic performance, stimulated antioxidant defenses, and decreased heavy-metal accumulation in roots, illustrating how biocontrol and contaminant detoxification can be integrated within the same management system [127]. More broadly, plant-associated microorganisms capable of metal sequestration, xenobiotic degradation, nutrient solubilization, siderophore production, and pathogen suppression represent particularly attractive tools for restoring contaminated agricultural soils.

### 4.4. Limitations of the Evidence Base

The evidence synthesized in this review is constrained by several methodological and contextual limitations that warrant cautious interpretation. A considerable proportion of the included studies employed laboratory-scale experiments or controlled microcosms, which, although valuable for mechanistic insights, may not fully capture the environmental complexity of field conditions. Soil heterogeneity, fluctuating hydrological regimes, and climate-driven variability are often incompletely represented, limiting the extrapolation of results to real-world agroecosystems.

Many studies investigated only binary combinations of metals and pesticides, whereas agricultural soils typically contain far more complex contaminant mixtures. The focus on simplified exposure scenarios may underestimate interactive effects that arise from multi-component systems. Further, heterogeneity in experimental design, exposure concentrations, and analytical protocols complicates cross-study comparisons. Reported contaminant levels frequently exceed environmentally realistic concentrations, reflecting a bias toward high-dose studies that may magnify toxicological responses.

The evidence base is also geographically skewed, being dominated by research from Asia (particularly China and India), with limited representation from Africa, South America, and Eastern Europe. This imbalance constrains the global generalisability of the findings, especially given region-specific differences in agricultural practices, soil properties, and climate regimes. Finally, relatively few studies examined long-term, chronic co-exposure effects or investigated the persistence and transformation of metal–pesticide complexes through seasons or cropping cycles. Such temporal gaps impede the development of comprehensive risk assessments.

The conclusions of this review should therefore be interpreted with appropriate caution, recognizing that variations in soil type, climatic conditions, exposure pathways, contaminant speciation, biological endpoints, and analytical sensitivity may influence outcomes. Nevertheless, by synthesizing a broad range of mechanistic, ecological, and agronomic evidence, this review provides a comprehensive overview of current understanding of how heavy metal–pesticide mixtures interact within agroecosystems and the broader implications for soil health, crop productivity, ecological integrity, and One Health risks.

### 4.5. Limitations of the Review Process

Additionally, heterogeneity in study designs, exposure metrics, endpoints, and statistical reporting precluded formal meta-analysis. This limitation was also evident during eligibility assessment: among the 788 publications subjected to extended audit, only 134 (17.0%) met the eligibility criteria directly, whereas 400 (50.8%) required full-text verification because metal–pesticide co-contamination could not be confirmed from the abstract, and 254 (32.2%) were excluded. The thematic composition of the final prioritized corpus further illustrates the limited depth of quantitative exposure evidence: only 28 of 165 publications (17.0%) were associated with risk assessment, 8 (4.8%) with dietary exposure, 4 (2.4%) with environmental monitoring, and 2 (1.2%) with edible tissues.

Consequently, the review relies primarily on narrative synthesis, limiting quantitative comparison of effect sizes and uncertainty across studies. Many health-risk assessments in the underlying literature use deterministic estimates based on contaminant concentrations, food consumption, body weight, and exposure frequency, which may not adequately capture population variability or parameter uncertainty. Probabilistic Monte Carlo simulations can provide distributions of exposure and risk, while sensitivity analysis can identify the variables that most strongly influence the resulting estimates. Where sufficient primary data are available, a two-dimensional Monte Carlo simulation can further separate population variability from parameter uncertainty and provide a more explicit characterization of uncertainty in mixture-risk assessment [63].

Finally, potential publication bias should be acknowledged, given the greater likelihood that studies reporting significant or positive contaminant effects are published and subsequently represented in the available evidence base.

### 4.6. Implications for Practice, Policy, and Future Research

The findings highlight an urgent need for integrated soil management strategies that acknowledge the interactive behavior of metals and pesticides and their potential transfer into food crops and the wider food chain. Agricultural practitioners should prioritize practices that minimize contaminant accumulation, such as precision pesticide application, reduced reliance on agrochemicals, and incorporate organic amendments that enhance sorption capacity and microbial resilience. The potential additive or synergistic effects reported for some metal–pesticide combinations underscore the importance of adopting biological and ecological remediation approaches with proven effectiveness in reducing contaminant mobility, crop uptake, and food-chain transfer.

Routine monitoring of metal–pesticide mixtures in soils, irrigation water, and crop tissues should become standard practice, particularly in high-risk regions characterized by intensive agriculture or wastewater irrigation. Monitoring programs should also consider edible plant tissues and, where relevant, animal-derived foods in order to better characterize dietary exposure. Results indicate that single-contaminant analyses may underestimate environmental and food-safety risks when mixture interactions are not considered.

Policymaking should shift from isolated-contaminant frameworks to cumulative-exposure regulation. Current legislation often sets separate limits for metals and for pesticides, overlooking the emergent risks posed by their co-occurrence. Regulatory bodies should consider developing mixture-based assessment approaches, guidance values, and integrated soil- and food-safety indicators that better reflect realistic exposure scenarios.

Stronger regulatory oversight of agrochemical manufacturing, wastewater reuse, and industrial emissions is warranted to prevent mixed contamination at source. Investment in national soil-monitoring networks and harmonized reporting systems would improve data availability, particularly in low- and middle-income countries where contamination risks are rising. Closer integration between soil-monitoring and food-control systems would also facilitate earlier identification of contaminants entering edible crops and food products. Furthermore, policy mechanisms such as subsidies, certification schemes, and financial incentives could accelerate the adoption of eco-remediation and low-input agricultural practices consistent with circular-economic principles.

Several critical research gaps remain. Long-term field studies are required to assess the persistence, transformation, and seasonal behavior of metal-pesticide complexes under realistic climatic and agronomic conditions. Prioritize the interactive effects of multi-component mixtures beyond binary pairs, using factorial designs and high-resolution analytical techniques to capture non-linear responses and their implications for contaminant uptake and residue formation in food crops.

Further work is needed to integrate climate-change projections into contaminant fate models, particularly regarding temperature-dependent kinetics, extreme rainfall events, and drought cycles. Advances in molecular biology and multi-omics approaches offer opportunities to unravel species-specific and community-level responses, thereby improving environmental and food-safety risk assessments. Greater attention should also be given to linking soil and plant biomarkers with measurable contaminant residues in edible tissues and dietary exposure estimates.

Lastly, the ecological safety of emerging remediation technologies, including nanoparticles, biochar–nanocomposites, and genetically enhanced microbes, must be evaluated comprehensively across trophic levels and time scales to ensure solutions do not generate new, unforeseen environmental or food-safety risks. Their effectiveness should, therefore, be assessed not only by contaminant removal or immobilization, but also by their ability to reduce residue transfer into food, preserve nutritional quality, and maintain long-term agroecosystem function.

## 5. Conclusions

The evidence reviewed indicates that heavy metal–pesticide co-occurrence is reported in agricultural and food-related systems, but the strength of evidence varies across study types and endpoints. Of the 165 publications retained in the final corpus, only a limited subset provided direct evidence of metal–pesticide co-exposure or interaction, while a larger proportion contributed co-occurrence, mechanistic, contextual, or supporting evidence.

Direct and moderate-strength evidence indicates that combined metal–pesticide exposure can modify contaminant mobility, plant uptake, oxidative stress responses, microbial processes, and toxicological outcomes. However, interactions that are synergistic, antagonistic, or additive depend strongly on contaminant identity, concentration, exposure duration, organism, matrix, and environmental conditions. These effects should, therefore, not be generalized beyond comparable experimental or environmental settings.

From a food-safety perspective, the most robust conclusions concern analytically confirmed co-occurrence in food-related matrices and studies evaluating dietary exposure or health-risk endpoints. Evidence that was directly linked to metal–pesticide interactions to increased contaminant accumulation in edible tissues remains more limited, highlighting the need for additional studies that simultaneously measure both contaminant classes, crop transfer, edible-tissue residues, and dietary exposure.

Climate-related effects on contaminant fate and toxicity should be interpreted according to evidence type. Model-based studies project changes in persistence, transport, leaching, and plant uptake under future climate scenarios, whereas experimental mesocosm, microcosm, controlled temperature, and field studies provide measured evidence of temperature- and hydrology-dependent changes in contaminant behavior and biological response. These effects remain strongly context dependent and should not be extrapolated beyond the conditions in which they were demonstrated.

Sustainable remediation strategies, including phytoremediation, biochar-based amendments, microbial approaches, and integrated plant–microbe–amendment systems, show potential to reduce contaminant mobility and crop uptake while preserving soil function. However, comparative effectiveness remains difficult to establish because contaminant combinations, environmental conditions, experimental designs, and reported endpoints vary widely.

Future research should, therefore, prioritize direct metal–pesticide co-exposure studies, long-term field validation, environmentally realistic contaminant mixtures, standardized reporting of interaction effects, and direct measurement of residues in edible tissues. Greater integration of soil monitoring, crop-residue analysis, dietary exposure assessment, and food-safety surveillance will be necessary to strengthen the evidence base and support risk management within One Health and sustainable-agriculture frameworks.

## Figures and Tables

**Table 1:** Integrated review scope, evidence coverage, methodological framework, and contribution of the present review.

Review Scope	Evidence Coverage	Methods	Integrated Contribution
Occurrence and co-distribution	Sources, mobility, co-detection, environmental distribution	PRISMA-informed screening; thematic classification	Integrates metal–pesticide co-contamination across agroecosystems
Interaction mechanisms and combined toxicity	Sorption, bioavailability, synergistic/antagonistic effects, plant and microbial responses	Distinction between co-detection, combined exposure, and demonstrated interactions	Separates simple co-occurrence from true interaction evidence
Food chain and health implications	Plant uptake, edible tissues, trophic transfer, food safety, human-health risk	Crop Uptake and Food Safety evidence domains	Links environmental contamination with food chain and One Health consequences
Climate-related effects	Drought, temperature, flooding, soil moisture, contaminant mobility and degradation	Dedicated Climate domain	Evaluates climate as a modifier of contaminant fate and effects
Sustainable mitigation	Phytoremediation, bioremediation, biochar, immobilization, microbial strategies	Dedicated Mitigation domain	Connects contaminant control with soil health and agroecosystem resilience
Evidence robustness	Screened, audited, and prioritized publication pool	Q85 prioritization with Q80/Q85/Q90 sensitivity analysis	Provides a transparent and reproducible evidence-prioritization framework

**Table 2:** Thematic categories, relevance domains and prioritization system.

Parameter	Number
Records retrieved	11,259
Include + Review	5157
Unique Top-200 evidence pool	1309
Records not retained for extended eligibility audit	521
Extended audit corpus	788
Excluded at eligibility audit	254
Eligible	450
Full-text check before ranking	84
Eligible + potentially eligible for ranking	534
Q85 thematic assignments	192
Q85 unique publications	165
Confirmed eligible in Q85 corpus	156
Final full-text checks	9

**Table 3:** Thematic categories and application domains.

Weight	Priority	Application Domain	Examples
7	Very high	Core exposure/transfer terms	Edible tissue; dietary exposure; food-chain transfer
6	High	Strong co-contamination/remediation terms	Co-contamination; mixture toxicity; bioremediation
5	High	Specific uptake/stress terms	Plant uptake; bioaccumulation; oxidative stress
4	Moderate	Supporting process terms	Translocation; bioavailability; rhizosphere
3	Moderate	General environmental processes	Sorption; rainfall; soil moisture
2	Low	Broad contextual terms	Fertilizer; plant growth; temperature

**Table 4:** Sensitivity analysis of percentile-based evidence prioritization.

Threshold	Thematic Assignments	Unique Publications	Q85 Retained (%)	Jaccard vs. Q85
Q80	249	193	100.0	0.855
Q85	192	165	100.0	1.000
Q90	132	116	70.3	0.703

**Table 5:** Category-specific ranking populations, Q85 relevance-score cutoffs, and retained thematic assignments.

Thematic Category	Candidate n	Q85 Threshold	Q85 Selected
Sources and Pathways of Heavy Metals and Pesticides in Agroecosystems	91	12	16
Crop Uptake, Translocation, and Bioaccumulation	104	11	27
Physicochemical Interactions and Contaminant Fate	97	11	31
Plant Physiological, Cellular, and Molecular Responses	159	14	41
Microbial Responses and Soil–Microbiome Processes	95	12	17
Food Safety, Dietary Exposure, and Food-Chain Transfer	155	19	26
Climate Modulation of Contaminant Fate and Biological Transfer	74	14	12
Sustainable Mitigation and Remediation Strategies	72	12	22
Total Thematic Evidence Assignments Across All Domains	—	—	192

**Table 6:** Representative Metal–Pesticide Co-exposure Pairs, Interaction Types, and Resulting Environmental or Biological Effects in Agroecosystems.

Metal–Pesticide Combination	System	Main Modifying Effect	Key Observation	Source
Cd + penflufen	Zebrafish	Increased pesticide persistence and bioaccumulation	Increased bioaccumulation and residue persistence	[40]
Cu + current-use pesticides	Rice	Altered root uptake and translocation	Enhanced root-to-shoot pesticide translocation	[46]
Cd + trichlorfon	*Brassica juncea*/water	Mutual influence on contaminant removal	Mutual inhibition of contaminant removal	[39]
Hg + chlorpyrifos	Soil–*Phaseolus vulgaris*	Co-contaminant uptake and remediation	Amendment-dependent co-contaminant removal	[38]
Cu + pesticide residues	Rice	Metal-mediated modification of pesticide transport	Cu altered pesticide translocation and root excretion	[46]

**Table 7:** Representative pesticide functional groups involved in complexation with divalent metal ions in soils.

Pesticide	Molecular Formula	Relevant Functional Groups	Simplified Chemical Structure/Binding Motif	Main Potential Metal-Binding Atoms	Relevance to Metal Complexation
Glyphosate	C_3_H_8_NO_5_P	Carboxyl, phosphonate, secondary amine	HOOC–CH_2_–NH–CH_2_–P(=O)(OH)_2_	Carboxylate O, phosphonate O, amine N	Strong multidentate ligand; can chelate Cu^2+^, Zn^2+^, Cd^2+^ and Pb^2+^ through O- and N-donor groups
Glufosinate	C_5_H_12_NO_4_P	Carboxyl, phosphinate, primary amine	HOOC–CH(NH_2_)–CH_2_–CH_2_–P(=O)(OH)–CH_3_	Carboxylate O, phosphinate O, amine N	Multiple donor sites permit coordination with divalent metal ions; complexation depends strongly on pH and metal concentration
2,4-D	C_8_H_6_Cl_2_O_3_	Carboxyl	2,4-Cl_2_–C_6_H_3_–O–CH_2_–COOH	Mainly carboxylate O	Deprotonated carboxylate can bind metal cations and participate in surface-associated metal bridging
MCPA	C_9_H_9_ClO_3_	Carboxyl	4-Cl-2-CH_3_–C_6_H_3_–O–CH_2_–COOH	Mainly carboxylate O	Metal coordination occurs mainly through the carboxylate group and may alter sorption and mobility
Atrazine	C_8_H_14_ClN_5_	Amino groups and triazine-ring N atoms	1,3,5-triazine ring bearing ethylamino and isopropylamino substituents	N donor atoms	Nitrogen atoms may participate in metal coordination, although binding is generally more condition-dependent than for glyphosate
Chlorpyrifos	C_9_H_11_Cl_3_NO_3_PS	Phosphorothioate, pyridyl N	Pyridyl–O–P(=S)(OEt)_2_	Pyridyl N and phosphorothioate-associated S/O atoms	Metal interactions may involve pyridyl N and phosphorothioate-associated donor atoms and are strongly dependent on environmental conditions

**Table 8:** Representative evidence of contaminant transfer and food-safety risk across plant- and animal-derived foods.

Food/System	Contaminants	Main Quantitative Evidence	Risk Implication	Source
Vegetables, Yamuna floodplain	Metals + OCPs	TF > 1 for Ni, Cr, Cu, and Zn in most samples	HI > 1 for most vegetables; HQ > 1 in children for radish and cauliflower	[69]
Rice, Hubei	Cd, As and other metals	Cd above soil limit in 89.1%; Cd and Ni above food limits in 58.7% and 52.2% of rice	Cd + As = 80.8% of non-carcinogenic risk	[65]
Lettuce	ZnO nanoparticles + imidacloprid	IMI biotransformation increased by 5.8–130%; Zn and IMI increased in shoots	Co-exposure enhanced accumulation and phytotoxicity	[66]
Market fruits and vegetables	18 highly toxic pesticides	4.96% positive; 1.57% above MRLs	Mean HI < 1 for adults and children	[70]
Raisins	Pesticide residues	23% contaminated by at least one pesticide	HI 0.001 adults; 0.006 children	[71]
Pistachios	Pesticides	0.015–0.064 mg kg^−1^	95th-percentile THQ 1.471 adults; 10.644 children	[72]
Rice, multiple origins	Metals + pesticides + mycotoxins	HI: 2.10 Pakistan; 1.86 India; 1.01 Iran; 0.98 Thailand	As or diazinon dominated depending on origin	[73]
Iranian rice	Metals + diazinon + mycotoxins	Maximum HI ≈5.86 adults; ≈11.94 children	Cr/OTA dominated; diazinon contribution low	[74]
Milk and cheese	Cd, Cu, Pb, Zn + DDT-related residues	Pb and DDD exceeded MRLs in all three areas	Pb HQ > 10; Cu and ΣDDT HQ > 1	[75]
Dried fish	Metals + pesticides	Pesticide HRI 0.013–0.16; metal THQ < 1	Metal carcinogenic risk remained relevant	[76]
*Sphoeroides* spp.	Metals + OCPs	20 OCPs and 7 metals detected	THQ > 1 at 120 g day^−1^; elevated lifetime cancer risk	[77]
Fish/crab, Indian estuary	Trace metals	Some Cr and Pb exceeded limits	Mean HI > 1 for children	[78]
Common fish, Dhaka	Heavy metals	APLI > 1 in all analyzed species	HI > 1 for adults	[79]
Shrimp	OCP residues	Residue reduction 48.8–60.3% after cooking	Post-processing HQ < 1	[80]
Honey/beebread	Pesticides	Detection: 25.4% beebread; 2.8% honey	Low human dietary risk; ecological concern for thiamethoxam	[81]
*Labeo rohita*	Pb + endosulfan	Tissue accumulation up to 1.7-fold higher; >65% damaged erythrocytes	Significant synergistic toxicity	[82]

**Table 9:** Representative sustainable mitigation strategies for metal, pesticide, and combined contamination in agroecosystems.

Mitigation Strategy	Target/System	Main Mechanism	Key Performance Indicator	Source
Rhizosphere bioaugmentation	Bensulfuron-methyl/maize	Root-exudate-assisted bacterial degradation	80.9% degradation; complete removal with *L*-malic acid	[111]
Microbial bioaugmentation	Epoxiconazole/soil–vegetable	*Pseudomonas*-mediated biodegradation	92.1% removed in 6 d; t½ reduced from 96.3 to 2.64 d	[112]
Bioaugmentation + adsorption/phytoremediation	Pentachlorophenol/wastewater	Bacterial degradation combined with adsorption or macrophytes	97% removal after 48 h; ~90% after 72 h	[113]
Microbial remediation/biocontrol	As/brinjal	Siderophore-mediated As immobilization + pathogen suppression	As(V) −74%; As(III) −88.1%	[114]
Plant + biochar + mycorrhiza	Hg + chlorpyrifos/soil	Combined phytoremediation and immobilization	Biochar–mycorrhiza combination—highest remediation performance	[38]
Porous biopolymer microspheres	Cu + hymexazol/rice soil	Immobilization + stimulation of beneficial microbiota	Root accumulation −18.46% Cu and −18.67% hymexazol	[115]
Zn-modified biochar/MgFe-LDH	Cd + atrazine/soil–maize	Adsorption, complexation and stabilization	Leaching −90.3% Cd and −89.7% atrazine	[116]
Microbial consortium	Chlorpyrifos + cypermethrin + lindane	Biosurfactant-assisted biodegradation	Near-complete chlorpyrifos/cypermethrin removal; lindane up to 93.6%	[117]
ZnO–TiO_2_ nanomaterial	Tomato bacterial disease	Antimicrobial action + induced plant defense	79.83% disease control	[118]
Myco-synthesized CuO nanoparticles	Potato fungal disease	Antifungal activity + plant defense stimulation	33.95% fungal inhibition; tuber yield increased up to 40%	[119]
Genistein botanical fungicide	*Alternaria solani*/tomato	ROS-mediated antifungal activity	EC50 6.53 µg mL^−1^; 48.1% protective and 42.5% therapeutic efficacy	[120]
Physcion botanical fungicide	*Penicillium choerospondiatis*	Membrane disruption + ROS induction	EC50 3.30 mg L^−1^	[121]
Novel antifungal LN18	*Monilinia fructicola*	Membrane disruption + ROS production	EC50 0.17 µg mL^−1^	[122]
ZIF-8 nano-antimicrobial	Rice bacterial leaf streak	Membrane/DNA damage + ROS induction	EC50 0.15 mg mL^−1^	[123]

## Data Availability

No new data were created or analyzed in this study. Data sharing is not applicable to this article.

## Supplementary Materials

The following supporting information can be downloaded at: https://www.mdpi.com/article/10.3390/foods15214012/s1, Table S1. Search Strategy Used for Each Database; Table S2: PRISMA 2020 Checklist.
