# Supplementary material for: Heavy Metal–Pesticide Co-Contamination Under Climate Change: A Systematic Review of Crop Uptake, Food-Chain Transfer, and Sustainable Mitigation
Source: Foods. 2026 Nov 9;15(21):4012. doi: 10.3390/foods15214012 (PMC13763321; doi:10.3390/foods15214012)
Supplement: Supplementary file 1 [file foods-15-04012-s001.zip › Supplementary File S1.pdf]

Supplementary. Search Strategy Details

Table S1. Search Strategy Used for Each Database

Database	Search Date	Search String (Boolean Logic)	Filters Applied
Web of Science Core Collection	30 December 2024	TS = ((“heavy metal*” OR “toxic metal*” OR cadmium OR lead OR copper OR arsenic OR “trace metal*”) AND (“pesticide*” OR “agrochemical*” OR “organophosphate*” OR “organochlorine*” OR atrazine OR chlorpyrifos OR paraquat) AND (“co-contamination” OR “co-contaminant*” OR “co-exposure” OR “combined pollution” OR “mixed contaminant*” OR “multi-contaminant*”) AND (“soil” OR “agricultural soil” OR rhizosphere OR “field soil” OR crop OR plant OR “soil microbiome” OR “soil enzyme*”) AND (“toxicity” OR “synergistic effect*” OR “interaction*” OR “oxidative stress” OR bioaccumulation OR sorption OR mobility OR “risk assessment”))	Timespan: 2000–2024; Language: English; Document type: Article
Scopus	30 December 2024	TITLE-ABS-KEY((“heavy metal*” OR cadmium OR lead OR arsenic OR chromium OR mercury OR nickel) AND (“pesticide*” OR “agrochemical*” OR atrazine OR chlorpyrifos OR “organochlorine pesticide*” OR “organophosphate pesticide*”) AND (“co-contamination” OR “combined pollution” OR “dual contamination” OR “co-exposure” OR “mixed pollution” OR “synergistic toxicity”) AND (“soil” OR “agricultural soil” OR “crop*” OR rhizosphere OR “soil microorganism*” OR “soil enzyme activity”) AND (“uptake” OR “translocation” OR “oxidative stress” OR “microbial activity” OR “ecotoxicity” OR “risk index”)))	Years: 2000–2024; Language: English; Source type: Journals
ScienceDirect	30 December 2024	(“heavy metal” OR cadmium OR lead OR arsenic) AND (“pesticide” OR “organophosphate” OR “organochlorine” OR atrazine OR chlorpyrifos) AND (“co-contamination” OR “co-exposure” OR “combined pollution” OR “multi-contaminant”) AND (“soil” OR “agricultural soil” OR “crop” OR plant OR rhizosphere OR “soil microbiome”) AND (“toxicity” OR “mechanism” OR “oxidative stress” OR bioavailability OR “food chain” OR “risk assessment”)	Research articles only; 2000–2024; English
PubMed	30 December 2024	((“Heavy Metals”[MeSH] OR cadmium OR lead OR arsenic OR copper) AND (“Pesticides”[MeSH] OR pesticide* OR atrazine OR chlorpyrifos OR “Organic Pollutants”) AND (“Environmental Exposure”[MeSH] OR “co-exposure” OR “mixed exposure” OR “co-contamination” OR “combined pollution”) AND (“Soil”[MeSH] OR soil OR plant* OR crop* OR “soil bacteria” OR “soil microbiota”)) AND (toxicity OR oxidative stress OR bioaccumulation OR sorption OR “risk assessment”)	Article type: Journal Article; Species: not restricted; English; 2000–2024
